# A Single Origin for Nymphalid Butterfly Eyespots Followed by Widespread Loss of Associated Gene Expression

**DOI:** 10.1371/journal.pgen.1002893

**Published:** 2012-08-16

**Authors:** Jeffrey C. Oliver, Xiao-Ling Tong, Lawrence F. Gall, William H. Piel, Antónia Monteiro

**Affiliations:** 1 Department of Ecology and Evolutionary Biology, Yale University, New Haven, Connecticut, United States of America; 2 Yale Peabody Museum of Natural History, Yale University, New Haven, Connecticut, United States of America; New York University, United States of America

## Abstract

Understanding how novel complex traits originate involves investigating the time of origin of the trait, as well as the origin of its underlying gene regulatory network in a broad comparative phylogenetic framework. The eyespot of nymphalid butterflies has served as an example of a novel complex trait, as multiple genes are expressed during eyespot development. Yet the origins of eyespots remain unknown. Using a dataset of more than 400 images of butterflies with a known phylogeny and gene expression data for five eyespot-associated genes from over twenty species, we tested origin hypotheses for both eyespots and eyespot-associated genes. We show that eyespots evolved once within the family Nymphalidae, approximately 90 million years ago, concurrent with expression of at least three genes associated with early eyespot development. We also show multiple losses of expression of most genes from this early three-gene cluster, without corresponding losses of eyespots. We propose that complex traits, such as eyespots, may have originated via co-option of a large pre-existing complex gene regulatory network that was subsequently streamlined of genes not required to fulfill its novel developmental function.

## Author Summary

Butterfly eyespots play an essential role in natural and sexual selection, yet the evolutionary origins of eyespots and of their underlying gene regulatory network remain unknown. By scoring phenotypes and wing expression of five genes in 399 and 21 nymphalid species, respectively, we tested when eyespots and expression of their associated genes evolved. We found that the origin of eyespots was concurrent with the origin of the gene expression patterns, approximately 90 million years ago. Following this event, many genes expressed in eyespot development were lost in some lineages without a corresponding loss of eyespots, indicating substantial evolution in the cluster of genes associated with eyespots. This finding suggests that complex traits such as butterfly eyespots may initially evolve by re-deploying pre-existing gene regulatory networks, which are subsequently trimmed of genes that are unnecessary in the novel context.

## Introduction

One of the most conspicuous novelties in the animal world, the eyespot, has received considerable attention regarding its underlying developmental gene regulatory network [1]–[8], but the origin of this “novel” complex trait remains unknown [9]. At least twelve genes are known to be expressed in the future eyespot centers of developing wings in at least one of two model species, *Bicyclus anynana* (Butler) and *Junonia coenia* Hübner [6], [7], [10], including the transcription factors *Antennapedia* (*Antp*), *spalt* (*sal*), *engrailed* (*en*), and *Distal-less* (*Dll*) and the trans-membrane receptor *Notch* [2], [4], [6], [7]. However, of these twelve genes expressed in focal centers, at least six (*Antp*, *sal*, *Dll*, *Notch*, *patched*, and *hedgehog*) are not expressed in all species with eyespots [7], [8]. This variation in gene expression among species suggests eyespots may not be homologous within Nymphalidae and prompts further examination into the evolutionary origins of eyespots.

Unraveling the origins of this complex trait requires a broad comparative framework where the trait and the genes associated with the trait are investigated simultaneously. If eyespots evolved multiple times within the Nymphalidae, the developmental gene regulatory networks governing their production may not necessarily be homologous. In contrast, if nymphalid eyespots arose once, the observed variation in gene expression argues for lineage-specific changes in the developmental pathway generating eyespots. Here we integrate morphological, phylogenetic, and developmental data to evaluate the untested assumption that nymphalid eyespots are homologous. We discover that from both morphological and developmental perspectives of homology [11]–[13], nymphalid eyespots and an associated gene cluster arose a single time, early in the evolution of the Nymphalidae. From this single origin, multiple losses of gene expression have occurred, suggesting a novel means in which complex traits originate: from an initial gene regulatory network co-option followed by streamlining of extraneous network elements.

## Results/Discussion

**Figure 1:**
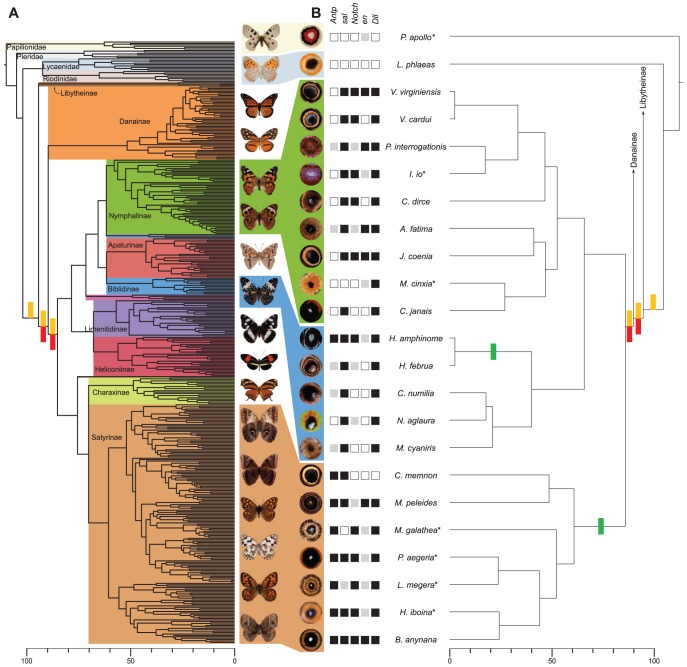
Origins of eyespots and associated gene expression. (A) Origin of eyespots inferred from 399 nymphalid and 29 outgroup species from phylogeny in [13]. (B) Origin of expression in eyespot centers inferred from gene expression profiles of 23 species. Presence or absence of expression of genes in future eyespot centers indicated by black and white boxes, respectively, and grey boxes indicate species/gene combinations for which expression data are unavailable. Green bars indicate two independent origins of eyespot-associated *Antp* expression. In both (A) and (B), divergence times (in millions of years) are from [13], [14]; red bars on the phylogeny indicate the possible locations of the single origin of eyespots, while gold bars indicate possible locations for the single origin of gene expression for *sal*, *Notch*, *Dll*, and possibly *en* in the eyespot centers. Asterisks (*) indicate species for which expression data are from [7], [8].

For a morphological assessment of homology we used Mayr's definition where “a feature is homologous in two or more taxa if it can be traced back to the same feature in the presumptive common ancestor.” (p. 45, [11]). If eyespots are homologous, there should be a single origin of this trait; in contrast, multiple origins of eyespots within the Nymphalidae would demonstrate that the traits are not homologous [14]. We reconstructed the history of eyespot evolution using adult wing characters for all 399 representative species included in a phylogenetic hypothesis of relationships of most nymphalid genera ([15] and Table S1). Ancestral state estimates indicate eyespots evolved once, twice, or three times in the history of the clade (Figure S1). To compare the likelihood of each of these scenarios, we performed likelihood ratio tests among the one, two, and three origin hypotheses. In all comparisons, the single-origin hypothesis provided a significantly better fit than the two- or three-origin hypotheses (Figure 1A and Table S2), demonstrating that nymphalid eyespots fulfill the phylogenetic homology criterion. Eyespots probably evolved close to the base of the Nymphalidae, after the split of the basal nymphalid subfamily Libytheinae and either before or after the split of the subfamily Danainae, during a relatively short 10 MY time window (red bars in Figure 1).

We next used gene expression profiles of 21 nymphalid and two outgroup species with eyespots (Figure S2) to determine if the gene regulatory networks associated with eyespot development are homologous within nymphalids and across butterfly lineages. Networks are considered homologous in two or more taxa if all the genes and their regulatory interactions can be traced back to the same network in the most recent common ancestor [12]. We addressed the first portion of this homology assessment by testing for gene expression in the most recent common ancestor of eyespot-bearing nymphalid species. When focal expression is reconstructed on the history of Nymphalidae, expression of *sal*, *Notch*, and *Dll* in future eyespot centers are all estimated to have arisen once, approximately 90 million years ago (Figure 1B). Ancestral state estimates for two genes, *Antp* and *en*, were ambiguous, with one or two origins of focal expression possible. Single-origin models had the highest likelihood for *en*, while *Antp* had a maximum likelihood estimate of two origins; likelihood ratio tests on these two genes show a better fit of the model espousing a two origins for *Antp* expression (green bars in Figure 1B), but cannot discern between one (identical to *sal*, *Notch*, and *Dll*) or two independent, more recent origins for *en* expression (Figure 1A, Figure S3, and Table S3). These results demonstrate that a majority of eyespot associated genes investigated here (*sal*, *Notch*, *Dll* and potentially *en*) have a single origin of expression in the eyespot centers, lending support to homology of the eyespot gene regulatory network across Nymphalidae. Eyespots in the closely related Lycaenidae (Figure S2V) and the more distantly related Papilionidae [8] do not express any surveyed genes at their center, suggesting an independent and developmentally distinct origin. Cursory examination of these and other butterfly lineages (Riodinidae and Pieridae) suggests that eyespots are rare in these clades and are more likely to have evolved multiple times recently, rather than once, early in the clades' evolution, as shown here for nymphalids; however, a more thorough comparative examination of eyespot evolution in these clades should be done in future.

**Figure 2:**
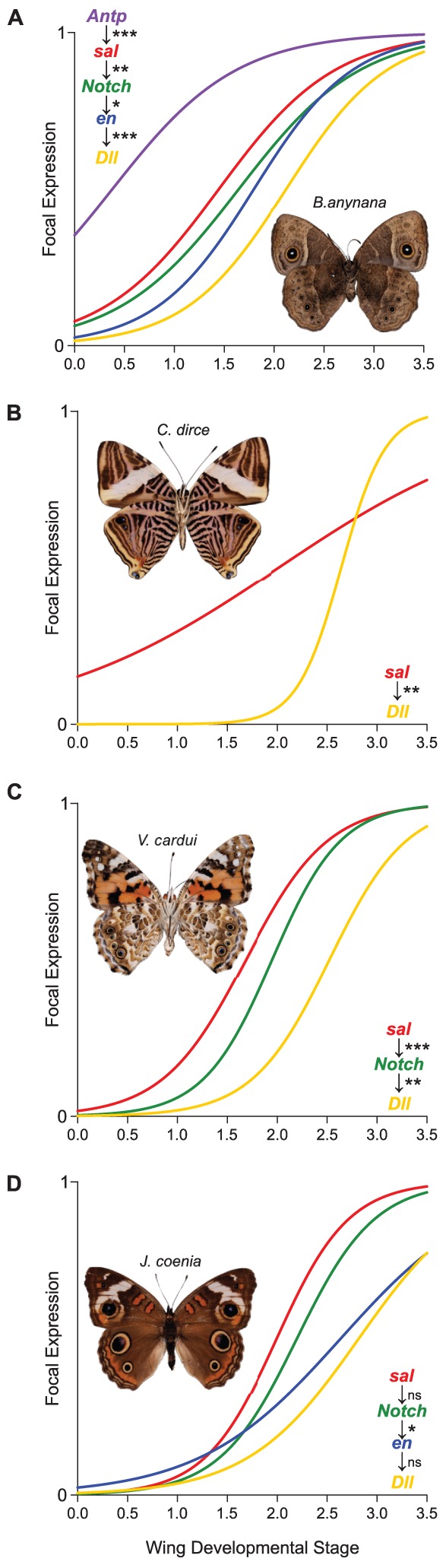
Onset of gene expression in future eyespot centers shows conserved temporal dynamics among nymphalids. Differences in relative timing of expression in the eyespot center between genes are consistent across (A) *B. anynana*, (B) *C. dirce*, (C) *V. cardui*, and (D) *J. coenia*. ***P<0.0001, **P<0.01, *P<0.05, ns = not significant at 0.05 level.

As a final assessment of homology, we tested whether the temporal patterns of gene expression within the network were conserved across taxa. Homologous gene regulatory networks should show similar relative temporal patterns of expression, while non-homologous networks would not be expected to show genes expressed in the same order across taxa [13]. Previous work in *J. coenia* and *B. anynana* suggested that eyespot focal genes are expressed in the following order: *Antp*→*sal*→*Notch*→*en*→*Dll* [5], [7], [16]. We measured establishment of focal expression in four taxa (*B. anynana*, *Colobura dirce* (Linnaeus), *Vanessa cardui* (Linnaeus), and *J. coenia*), spanning approximately 85 million years of divergence, and tested for conservation in the relative timing of expression in the future eyespot centers. Conserved timing of focal expression was highly supported in *B. anynana*, *C. dirce*, and *V. cardui* (Figure 2 and Figure S4). Although two of three comparisons in *J. coenia* were not significant, the relative timing of focal expression was qualitatively consistent with timing observed in the other three taxa. Similar patterns of conserved temporal dynamics were observed in co-stainings of pairs of genes in additional species (Figure S5). This conservation of temporal dynamics of gene expression in future eyespot centers further supports the hypothesis that the regulatory networks of eyespots are homologous.

The widespread expression of *Distal-less* and *spalt* in future eyespots of nymphalids suggests a conserved and functional role of these transcription factors in wing pattern development. *Dll* and *sal* were expressed in the future eyespot centers of all but one and two surveyed nymphalid species, respectively (Figure 1 and Figure S2). *Sal* expression is also associated with non-eyespot patterns in two species: in *Consul fabius* (Cramer), *sal* is expressed in larval wing discs in locations where crescent-shaped patterns develop (Figure S2F); in *Siproeta stelenes* (Linnaeus), wing expression of *sal* is associated with patches of black scales that develop on the ventral hind wings (Figure S2R).

Similarly, black patches of scales are associated with pupal stage expression of *Dll* and *sal* in *B. anynana* and with *sal* expression in the distantly related butterfly *Pieris rapae* (Linnaeus) (Pieridae) [6], [13]. Recent transgenic experiments suggest a functional role of *Dll* and *sal* in black scale development in *B. anynana* during the pupal stages of development (X. Tong, in review). Functional and comparative expression data together suggest that *Dll* and *sal* may have had a prior role in wing color pattern development, before they became co-opted into the eyespot center's regulatory network. The putative previous function of *Dll* and *sal* in color patterning wings, combined with the novel genetic background provided by other co-opted genes, may have facilitated the rapid appearance of an eyespot pattern.

With the exception of *Antp*, genes initially associated with eyespots demonstrate clear evidence of evolutionary lability. The genes estimated to have evolved concurrently with eyespots (*sal*, *Notch*, *Dll*, and possibly *en*) are not expressed in all future eyespot centers (Figure 1B and Figure S3). Previous studies have shown how loss of gene expression in complex regulatory networks is associated with the loss of a trait [17], but nymphalid butterflies show a pattern of network reduction without concurrent losses of eyespots. Although there is variation in the number of genes expressed in different species and the number of rings of colors in adult eyespots, we found no relationship between the two quantities (F_1,5_ = 1.56, *p* = 0.267). This widespread variation among species in the number of genes expressed during eyespot development suggests some elements of the gene regulatory network, in some species, either no longer play an essential role in eyespot development, or never played a role in the first place.

**Figure 3:**
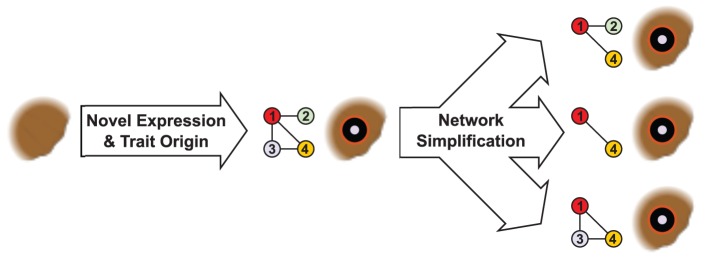
Regulatory network simplification in a complex trait. Following the origin of a complex trait and its underlying developmental gene regulatory network, genes that are non-functional or unnecessary may be subsequently removed from the network (genes 2 and 3), without eliminating the trait. Genes expressed in homologous traits of all taxa may represent a ‘core network’ of regulatory elements (genes 1 and 4) that are necessary for the development of the novel trait.

The correspondence of eyespot origins with an origin of expression of at least three of the five genes examined (Figure 1) is consistent with the hypothesis that eyespots originated from a gene network co-option event (Figure 3) [9]. This hypothesis posits a complex gene regulatory network involved in differentiating some other trait in a butterfly's body became expressed, in its entirety, in the future eyespot centers, and was subsequently rewired to generate the novel eyespot patterns. Subsequent network simplification is likely to happen when genes co-opted into the novel context are not functional in producing the novel trait. Loss of gene expression in the eyespot context may happen once network genes or their cis-regulatory elements duplicate, allowing the sub-functionalization and specialization of each copy for a different function [18]. This process of duplication and specialization provides for losses of expression in novel contexts (e.g. the eyespot), while expression is retained in the original context. Alternatively, genes from the original network may be secondarily co-opted to function in the development of the novel trait due to their fortuitous expression there. If different lineages undergo different paths of secondary co-option, this mechanism may provide an explanation for the phenomenon of developmental system drift, where networks diverge between lineages despite conservation of the final phenotype [19].

The relevance of this co-option hypothesis to eyespot evolution requires additional verification from multiple lines of evidence. First, it will be key to discover the identity and interactions of all genes in the eyespot developmental network, ideally exploiting comparative transcriptomic approaches. This may allow identification of putative ancestral gene regulatory networks originally involved in the co-option event. The existence of ‘source ancestral networks’ would provide additional evidence against the alternative hypothesis that the eyespot developmental network was built gradually, gene by gene. Transcriptomic data for eyespot-bearing species in the lycaenids and riodinids, which together form the sister group to Nymphalidae, may demonstrate a completely independent origin of the eyespot gene regulatory network or identify homologous parts of the eyespot network shared with nymphalids. Functional gene knock-downs that show parallel effects in similar clusters of target genes in more than one developmental context would also support the co-option scenario. In addition, *cis*-regulatory elements of genes co-opted as part of a larger network to the eyespot location should be pleiotropic and drive gene expression in the eyespots and in the original developmental context, whereas no such pleiotropic elements are expected if the eyespot gene regulatory network originated *de novo* [9]. Finally, comparative functional data will be necessary to understand how variation in eyespot gene regulatory networks translates to variation in morphology among butterfly species.

In summary, this study highlights the utility of the comparative approach in understanding the origins and evolution of complex traits. The differences in gene expression in eyespot centers among nymphalid species suggest considerable cryptic developmental variation in a homologous trait. This type of broad comparative survey should prove useful in identifying candidates for future functional studies within and across taxa: genes expressed in all or a majority of species likely play a necessary role in the development of a complex trait and should be the primary targets of functional experiments. Future comparative work in other systems will allow for additional tests of the co-option hypothesis, to determine how often complex traits originate via bursts of complexity in gene expression, followed by genetic streamlining of unnecessary elements.

## Materials and Methods

### Gene Expression

We dissected final instar larval wing discs from captive reared individuals and stained for gene products using the protocol described in [20]. *Bicyclus anynana* larvae were collected from the Yale colony established from Malawi. *Vanessa virginiensis* (Drury), *Polygonia interrogationis* (Fabricius), and *Lycaena phlaeas* (Linnaeus) larvae were collected in New Haven, CT. *Vanessa cardui* and *Danaus plexippus* (Linnaeus) larvae were purchased from Educational Science (League City, TX, USA). *Junonia coenia* larvae were provided by L. Grunert and H.F. Nijhout at Duke University. Larvae of all other species were obtained from The Butterfly Farm at Costa Rica Entomological Supply (La Guacima, Alajuela, Costa Rica) and from surrounding farms. Wings were stained for gene products of *Antp* (4C3 mouse monoclonal anti-*Antp* at 1∶400 concentration; Developmental Studies Hybridoma Bank), *sal* (GP66-1 guinea pig polyclonal anti-*sal* at 1∶20000), *Notch* (C17.9C6-s mouse monoclonal anti-*Notch* at 1∶20), *en* (4F11 mouse monoclonal anti-*en* at 1∶5, a gift from Nipam Patel), or *Dll* (rabbit polyclonal anti-*Dll* at 1∶200, a gift from Grace Boekhoff-Falk). We used donkey anti-mouse (Jackson Immunoresearch #715-095-150), goat anti-guinea pig (Molecular Probes #A11076), and goat anti-rabbit (Molecular Probes #T-2767) secondary antibodies at a concentration of 1∶200. All wings were mounted with ProLong Gold (Invitrogen, Carlsbad, CA, USA) and images of gene expression were captured on a Nikon 90i microscope using the NIS-Elements software (Nikon Instruments, Melville, NY, USA). To confirm that focal expression was not absent due to a failed immunostaining, we looked for presence of gene expression in other areas of the wing: *Dll* – along the margin and in the mid-line of most wing compartments; *en* – in the posterior compartment; *Notch* – along the veins; and by co-staining wings in the same session with those of *B. anynana* where focal expression is present for all genes. These antibodies are cross-reactive outside the Lepidoptera suggesting that the targeted protein epitopes are extremely conserved [21]–[24]. In addition, those antibodies raised against *Antp*, *sal*, and *Dll*, are polyclonal, further suggesting that absence of expression of these genes is unlikely to be due to molecular evolution of the epitope sequence as there are many possible epitopes that can be targeted by the antibody. All images will be archived at http://www.lepdata.org/monteiro/lepdata.html. Expression data for seven additional taxa (*Inachus io*, *Melitaea cinxia*, *Melanargia galathea*, *Pararge aegeria*, *Lasiommata megera*, *Heteropsis iboina*, *Parnassius apollo*) were taken from previously published works [7], [8].

### Origins of Eyespots

We reconstructed ancestral states of eyespots on the current estimate of nymphalid relationships and divergence times, and included all 29 Papilionoidea species selected as outgroups in the same phylogenetic estimate [15]. The outgroup species include representatives of all three subfamilies of Riodinidae and six of the seven families of Lycaenidae; these two families form the sister clade to the Nymphalidae. Each species was scored for presence or absence of eyespots at any location in adult wings. Eyespots were scored as any wing pattern element that (1) was roughly circular or oval and contained at least two concentric rings of color or (2) had a central pupil and a disc of color around it. All species were scored from the Yale Peabody Entomology Museum's image archive (http://www.lepdata.org/monteiro/lepdata.html). Ancestral state estimation was performed in Mesquite [25], using a two-parameter asymmetrical model of evolution, to allow different rates in gains versus losses of eyespots. Values for rate parameters were simultaneously optimized with ancestral state estimations. Divergence times within the nymphalid clade are from [15], while divergence times among families are from [26].

To test among one, two, or three origin hypotheses for the evolution of eyespots, we used BayesTraits [27] to compare likelihoods among hypotheses. In total, we compared six different models of evolution by calculating the likelihood of each model by fixing states at ancestral nodes corresponding to each hypothesis (Figure S1 and Table S2). Models were considered significantly different if log likelihood values differed by two or more log likelihood units [28].

### Origins of Eyespot Focal Gene Expression

Based on estimated nymphalid relationships [15] and relationships among butterfly families [26], we estimated the ancestral state of eyespot-associated focal expression using 21 nymphalid species, one lycaenid species, and one papilionid species (all of which have eyespots on adult wings) for which expression data were available. Divergence times between two pairs of species (*Vanessa cardui*/*V. virginiensis* and *Hamadryas amphinome* (Linnaeus)/*H. februa* (Hübner)) were not available in [15], so we based divergence times on cytochrome oxidase subunit I mitochondrial DNA sequences available from GenBank and the Barcode of Life Database, using an estimate of 2.3% sequence divergence expected per million years [29]. The two *Vanessa* species differed, on average, by 5.45%, yielding a divergence time of 2.37 mya, and the average pairwise difference between the two *Hamadryas* species was 5.71%, for a divergence time of 2.48 mya. The expression data include those presented in this study, as well as expression data for *Antp*, *sal*, *Notch*, and *Dll* for seven additional species from [7], [8]. We scored each gene for each species for presence or absence of focal expression in future eyespot centers (Figure S2). We estimated ancestral states for each gene separately in Mesquite [25], using a unique two-parameter model for each gene. As with analyses of morphological data, rate parameter values were simultaneously optimized with ancestral state estimations. The expression of three genes, *sal*, *Notch*, and *Dll*, was unambiguously reconstructed as evolving once, while two genes, *Antp* and *en*, required explicit tests of origins to distinguish between one or two origin hypotheses.

To distinguish among models of evolution for focal expression of *Antp* and *en*, we again used likelihood ratio tests in BayesTraits [27]. Fixing states at ancestral nodes corresponding to each hypothesis, we calculated model likelihoods and compared the log likelihoods among models (Figure S3 and Table S3). We rejected models that differed by two or more log likelihood units from the best fit model [28].

### Relative Timing of Eyespot Focal Gene Expression

Developmental stages of wing discs of *B. anynana*, *C. dirce*, *V. cardui*, and *J. coenia* were measured using the protocol of [5]. Wing compartments for each disc were recorded as having no focal expression (0) or focal expression (1) (Figure S6). Only those compartments that consistently displayed eyespots in adult wings were included for subsequent timing analyses. This resulted in the following compartments being analyzed: nine in *B. anynana* (forewing M_1_ and Cu_1_ and hindwing Rs, M_1_, M_2_, M_3_, Cu_1_, Cu_2_ and Pc); seven in *C. dirce* (forewing R_5_ and M_1_ and hindwing Rs, M_1_, M_2_, M_3_, and Cu_1_); six in *V. cardui* (forewing R_5_ and M_1_ and hindwing M_1_, M_2_, M_3_, and Cu_1_); and four in *J. coenia* (forewing M_1_ and Cu_1_ and hindwing M_1_ and Cu_1_). We first combined data across wing compartments for each species and analyzed data for each species without regard to compartment identity (e.g. data from all four compartments of *J. coenia* were combined in a single matrix of developmental stage and focal expression). Then, within each species, we fit a logistic curve to our data, where developmental stage was the predictor of focal expression. Data for each gene were modeled separately. To determine if timing of focal expression differed between individual genes, we used a method adapted from mRNA expression studies [30], which presents a null hypothesis of identical temporal expression between a pair of genes. The observed data from each gene are then a noisy representation of a single underlying relationship between developmental stage and focal gene expression. The difference in the temporal expression profiles between two genes is measured as the difference in the areas under each genes' logistic expression curve, δ_obs_. To assess significance of this difference, we compared δ_obs_ to a distribution of δ generated via bootstrapping from the observed data. Briefly, to compare the temporal expression profile of gene A to gene B, we first calculated δ_obs_. We then generated a distribution of differences (δ_null_) based on curves fitted to bootstrapped data, based on original sampling efforts of gene A and gene B. These bootstrapped samples are gene *A*, which is based on n_A_/2 samples observed for gene A and n_A_/2 samples observed for gene B, and gene *B*, based on n_B_/2 samples observed for gene A and n_B_/2 samples observed for gene B, where n_A_ and n_B_ are sample sizes for gene A and gene B, respectively. Under the null hypothesis, δ_obs_ should fall within the bounds of the δ_null_ distribution. We performed the following comparisons, based on 10,000 bootstrap replicates in each case: *B. anynana*: *Antp* vs. *sal*, *sal* vs. *Notch*, *Notch* vs. *en*, and *en* vs. *Dll*; *C. dirce*: *sal* vs. *Dll*; *V. cardui*: *sal* vs. *Notch* and *Notch* vs. *Dll*; *J. coenia sal* vs. *Notch*, *Notch* vs. *en*, and *en* vs. *Dll*. Curve-fitting and bootstrapping analyses were performed in the R software package [31].

### Correlation between Network Complexity and Eyespot Complexity

We tested for a correlation between the number of genes expressed in the eyespot center (network complexity) and the maximum number of different colored rings in adult eyespot pattern (trait complexity) for six nymphalid species for which complete staining profiles were available (Figure 1B). We used linear regression in the R software package [31] to determine if network complexity (number of genes) predicted eyespot complexity (number of rings).

## Supporting Information

Figure S1 Schematic of relationships used for likelihood ratio tests of eyespot origins within Nymphalidae. Analyses conducted on tree of 399 nymphalid species+29 outgroup species (in this figure, clades of each nymphalid subfamily and outgroup family are collapsed for ease of viewing). Letters at nodes indicate nodes used for fixing ancestral states in likelihood ratio tests (see Table S2). All clades except Libytheinae and Calinaginae include at least one species with eyespots on adult wings.
(PDF)

Figure S2 Gene expression profiles of 21 nymphalid species and 3 outgroup species. Gene expression in larval wing discs of (A) *Tithorea tarricina* hindwings, (B) *Danaus plexippus* forewings, (C) *Morpho peleides* forewings, (D) *Caligo memnon* forewings, (E) *Bicyclus anynana* hindwings, (F) *Consul fabius* hindwings, (G) *Hypna clytemenstra* forewing, (H) *Dryadula phaetusa* hindwings, (I) *Hamadryas amphinome* hindwings, (J) *Hamadryas februa* hindwings, (K) *Catonephele numilia* hindwings, (L) *Nessaea aglaura* hindwings, (M) *Myscelia cyaniris* hindwings, (N) *Vanessa virginiensis* forewings, (O) *Vanessa cardui* forewings, (P) *Polygonia interrogationis* hindwings, (Q) *Colobura dirce* hindwings, (R) *Siproeta stelenes* hindwings, (S) *Anartia fatima* hindwings, (T) *Chlosyne janais* hindwings, (U) *Junonia coenia* forewings, and outgroups (V) *Lycaena phlaeas* (Lycaenidae) forewings, (W) *Pieris rapae* (Pieridae) forewings, (X) *Papilio anchisiades* (Papilionidae) hindwings. Expression in eyespot centers is indicated by plus signs (‘+’). Genes that displayed no elevated expression in future eyespot centers are indicated by minus signs (‘−’).
(PDF)

Figure S3 Relationships among taxa used for inferring history of gene expression in future eyespot centers. Divergence times (in millions of years) within Nymphalidae from [13] and divergence times among families from [14]. Numbered nodes were used for fixing ancestral states in likelihood ratio tests (see Table S3). Expression for each gene indicated as: (−) no central expression, (+) central expression; taxon/gene combinations missing symbols indicate data not available. Asterisks (*) indicate species for which expression data are from [7], [8].
(PDF)

Figure S4 Temporal dynamics of gene expression in future eyespot centers of four nymphalid species. Graphs show the relationship between wing developmental stage and eyespot central expression (present vs. absent). Lines show best-fit logistic curves for each gene and sizes of points indicate relative number of samples observed at each developmental stage. See Figure S6 for central expression category examples.
(PDF)

Figure S5 Co-stains showing temporal differences in expression of pairs of genes in the eyespot centers. (A–C) *Antp* before *sal*; (D–E) *Antp* before *dll*; (F–I) *sal* before *Notch*; (J) *sal* before *en*; (K–L) *sal* before *Dll*; (M–N) *Notch* before *Dll*; and (O–P) *en* before *Dll*.
(PDF)

Figure S6 Eyespot central expression categories for temporal expression analyses. Images show anti-Sal antibody stains in the Cu_1_ compartment of *B. anynana* hindwings. Gene expression was categorized as zero (central expression absent) when no pattern of up-regulation was evident (A) or when expression was only detected in the midvein area (B). Gene expression was categorized as one (central expression present), when a clear cluster of central cells was detected, either in the presence (C) or absence (D) of expression in the midvein area.
(PDF)

Table S1 Data matrix for presence or absence of eyespots in 399 nymphalid taxa and 17 outgroup species.
(DOC)

Table S2 Model comparisons for the number of eyespot origins in Nymphalidae. Node states refer to ancestral state (0 = eyespots absent and 1 = eyespots present) assigned to nodes as lettered in Figure S1. Differences in log likelihoods are relative to the best-fit model of a single origin, after divergence of Danainae; bold ΔlnL values indicate models providing a significantly worse fit than the single origin model.
(DOC)

Table S3 Model comparisons of expression evolution of *en* and *Antp* in eyespot centers. Node states refer to ancestral state (0 = no central expression and 1 = expression in future eyespot centers) assigned to nodes as numbered in Figure S3. Differences in log likelihoods are relative to the best-fit model for each gene (the single origin model in *en* and the two origin, recent gain in Biblidinae model in *Antp*); significantly worse models are indicated by bold ΔlnL values.
(DOC)
