# Supplementary material for: A Single Origin for Nymphalid Butterfly Eyespots Followed by Widespread Loss of Associated Gene Expression
Source: PLoS Genet. 2012 Aug 16;8(8):e1002893. doi: 10.1371/journal.pgen.1002893 (PMC3420954; doi:10.1371/journal.pgen.1002893)
Supplement: Figure S1 — Schematic of relationships used for likelihood ratio tests of eyespot origins within Nymphalidae. Analyses conducted on tree of 399 nymphalid species+29 outgroup species (in this figure, clades of each nymphalid subfamily and outgroup family are collapsed for ease of viewing). Letters at nodes indicate nodes used for fixing ancestral states in likelihood ratio tests (see Table S2). All clades except Libytheinae and Calinaginae include at least one species with eyespots on adult wings. (PDF) [file pgen.1002893.s001.pdf]

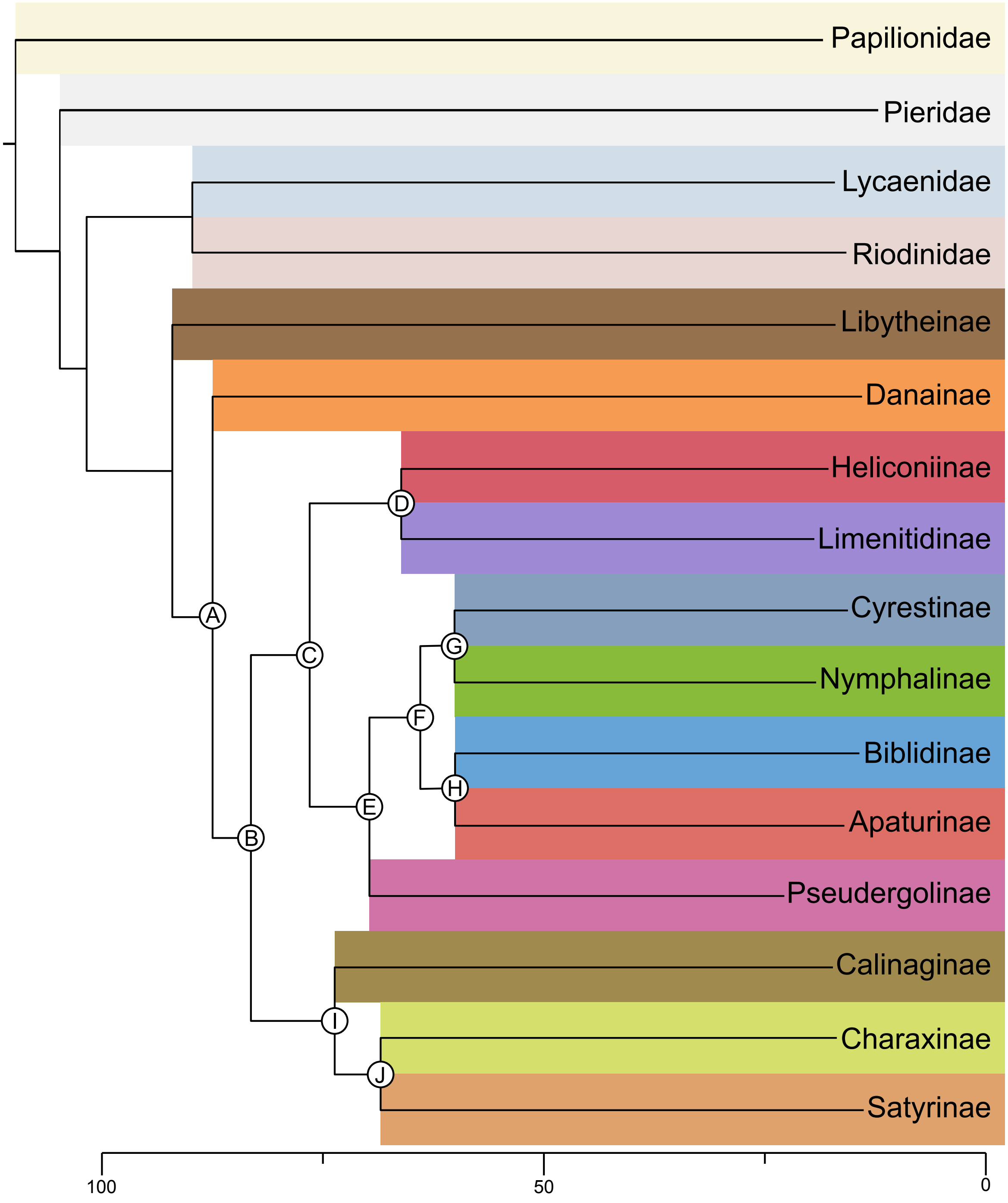

Figure S1. Schematic of relationships used for likelihood ratio tests of eyespot origins within Nymphalidae. Analyses conducted on tree of 399 nymphalid species + 29 outgroup species (in this figure, clades of each nymphalid subfamily and outgroup family are collapsed for ease of viewing). Letters at nodes indicate nodes used for fixing ancestral states in likelihood ratio tests (see Table S2). All clades except Libytheinae and Calinaginae include at least one species with eyespots on adult wings.
