# Supplementary material for: A Single Origin for Nymphalid Butterfly Eyespots Followed by Widespread Loss of Associated Gene Expression
Source: PLoS Genet. 2012 Aug 16;8(8):e1002893. doi: 10.1371/journal.pgen.1002893 (PMC3420954; doi:10.1371/journal.pgen.1002893)
Supplement: Figure S2 — Gene expression profiles of 21 nymphalid species and 3 outgroup species. Gene expression in larval wing discs of (A) Tithorea tarricina hindwings, (B) Danaus plexippus forewings, (C) Morpho peleides forewings, (D) Caligo memnon forewings, (E) Bicyclus anynana hindwings, (F) Consul fabius hindwings, (G) Hypna clytemenstra forewing, (H) Dryadula phaetusa hindwings, (I) Hamadryas amphinome hindwings, (J) Hamadryas februa hindwings, (K) Catonephele numilia hindwings, (L) Nessaea aglaura hindwings, (M) Myscelia cyaniris hindwings, (N) Vanessa virginiensis forewings, (O) Vanessa cardui forewings, (P) Polygonia interrogationis hindwings, (Q) Colobura dirce hindwings, (R) Siproeta stelenes hindwings, (S) Anartia fatima hindwings, (T) Chlosyne janais hindwings, (U) Junonia coenia forewings, and outgroups (V) Lycaena phlaeas (Lycaenidae) forewings, (W) Pieris rapae (Pieridae) forewings, (X) Papilio anchisiades (Papilionidae) hindwings. Expression in eyespot centers is indicated by plus signs (‘+’). Genes that displayed no elevated expression in future eyespot centers are indicated by minus signs (‘−’). (PDF) [file pgen.1002893.s002.pdf]

Figure S2. Gene expression profiles of 21 nymphalid species and 3 outgroup species. Gene expression in larval wing discs of (A) *Tithorea tarricina* hindwings, (B) *Danaus plexippus* forewings, (C) *Morpho peleides* forewings, (D) *Caligo memnon* forewings, (E) *Bicyclus anynana* hindwings, (F) *Consul fabius* hindwings, (G) *Hypna clytemenstra* forewing, (H) *Dryadula phaetusa* hindwings, (I) *Hamadryas amphinome* hindwings, (J) *Hamadryas februa* hindwings, (K) *Catonephele numilia* hindwings, (L) *Nessaea aglaura* hindwings, (M) *Myscelia cyaniris* hindwings, (N) *Vanessa virginiensis* forewings, (O) *Vanessa cardui* forewings, (P) *Polygonia interrogationis* hindwings, (Q) *Colobura dirce* hindwings, (R) *Siproeta stelenes* hindwings, (S) *Anartia fatima* hindwings, (T) *Chlosyne janais* hindwings, (U) *Junonia coenia* forewings, and outgroups (V) *Lycaena phlaeas* (Lycaenidae) forewings, (W) *Pieris rapae* (Pieridae) forewings, (X) *Papilio anchisiades* (Papilionidae) hindwings. Expression in eyespot centers is indicated by plus signs ('+'), while genes which displayed no elevated expression in future eyespot centers are indicated by minus signs ('−'). Numbers indicate sample size.

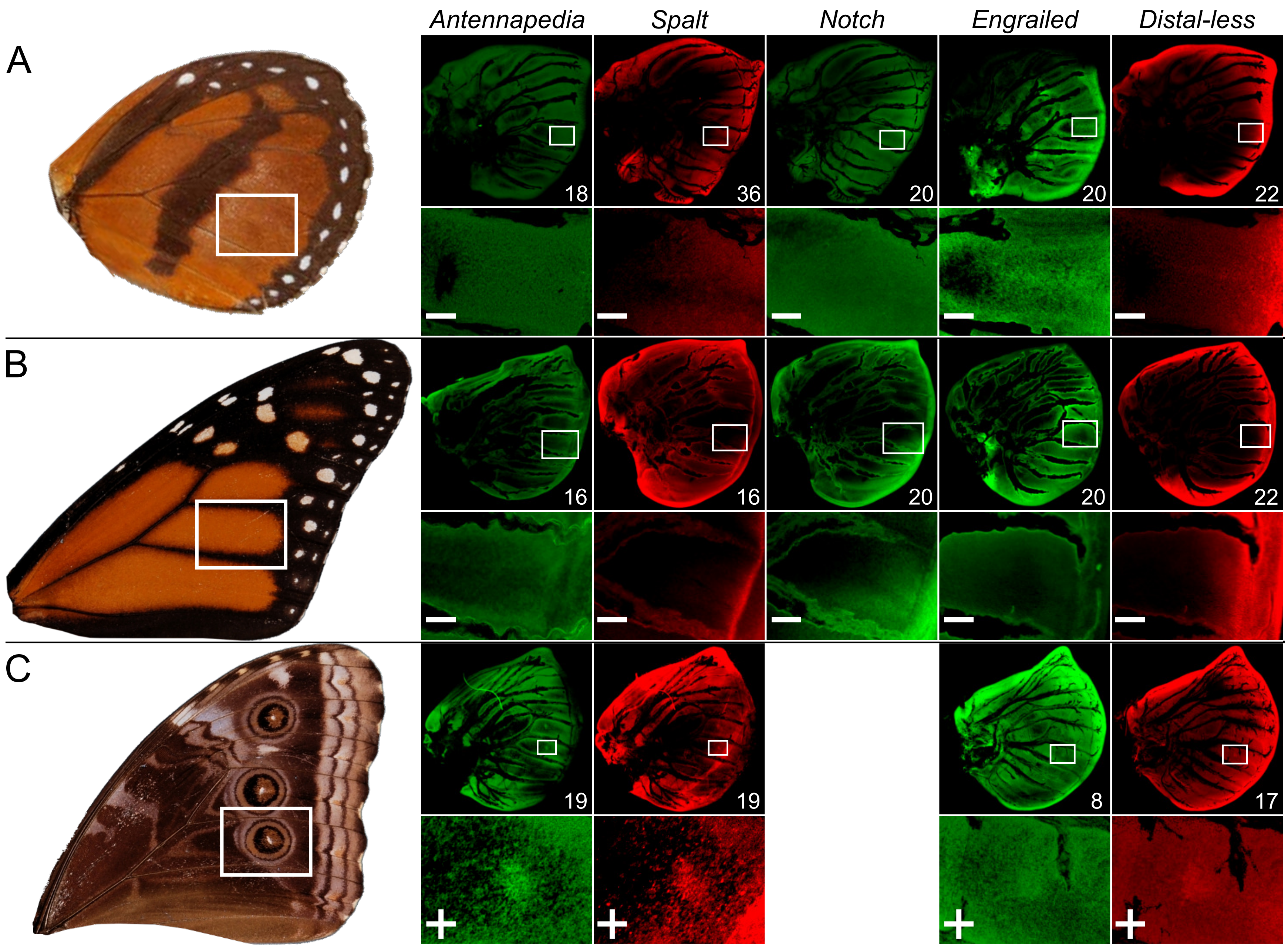

Antennapedia
Spalt
Notch
Engrailed
Distal-less
A
18
36
20
20
22
B
16
16
20
20
22
C
19
19
8
17

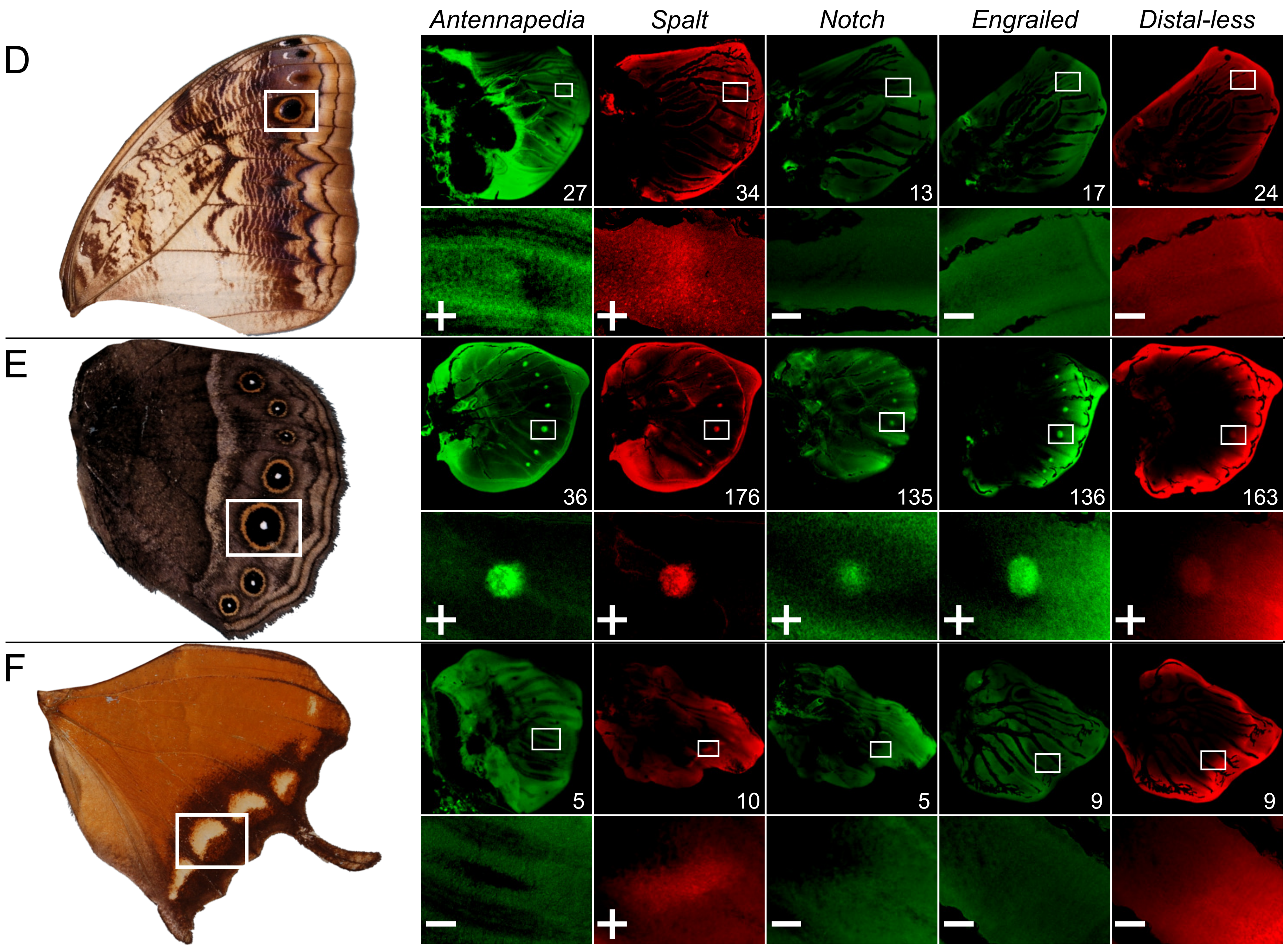

D
E
F
Antennapedia
Spalt
Notch
Engrailed
Distal-less
27
34
13
17
24
36
176
135
136
163
5
10
5
9
9

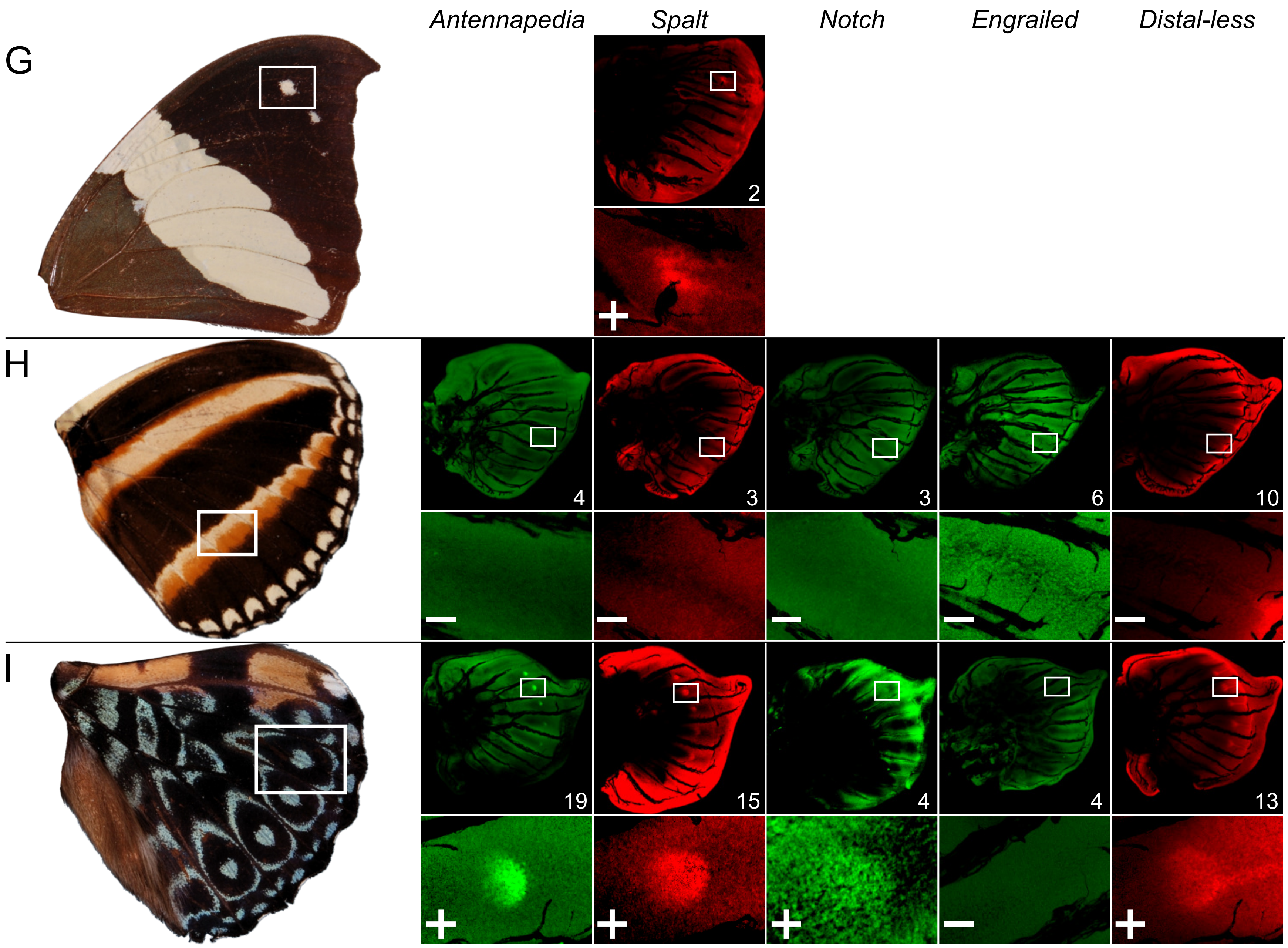

Antennapedia
Spalt
Notch
Engrailed
Distal-less
G
2
+
H
4
3
3
6
10
−
−
−
−
−
I
19
15
4
4
13
+
+
+
−
+

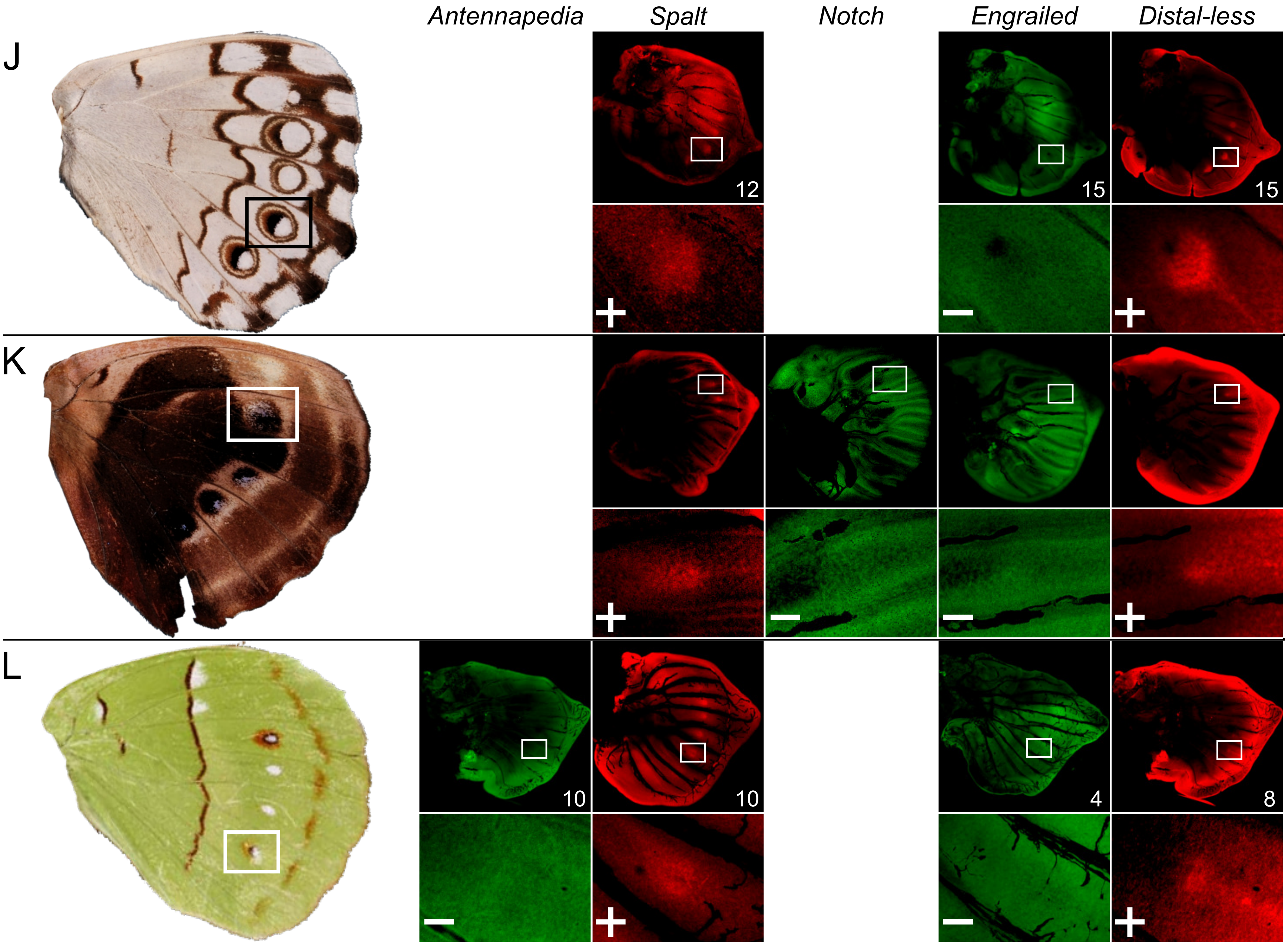

Antennapedia
Spalt
Notch
Engrailed
Distal-less
J
12
15
15
+
−
+
K
+
−
−
+
L
10
10
4
8
−
+
−
+

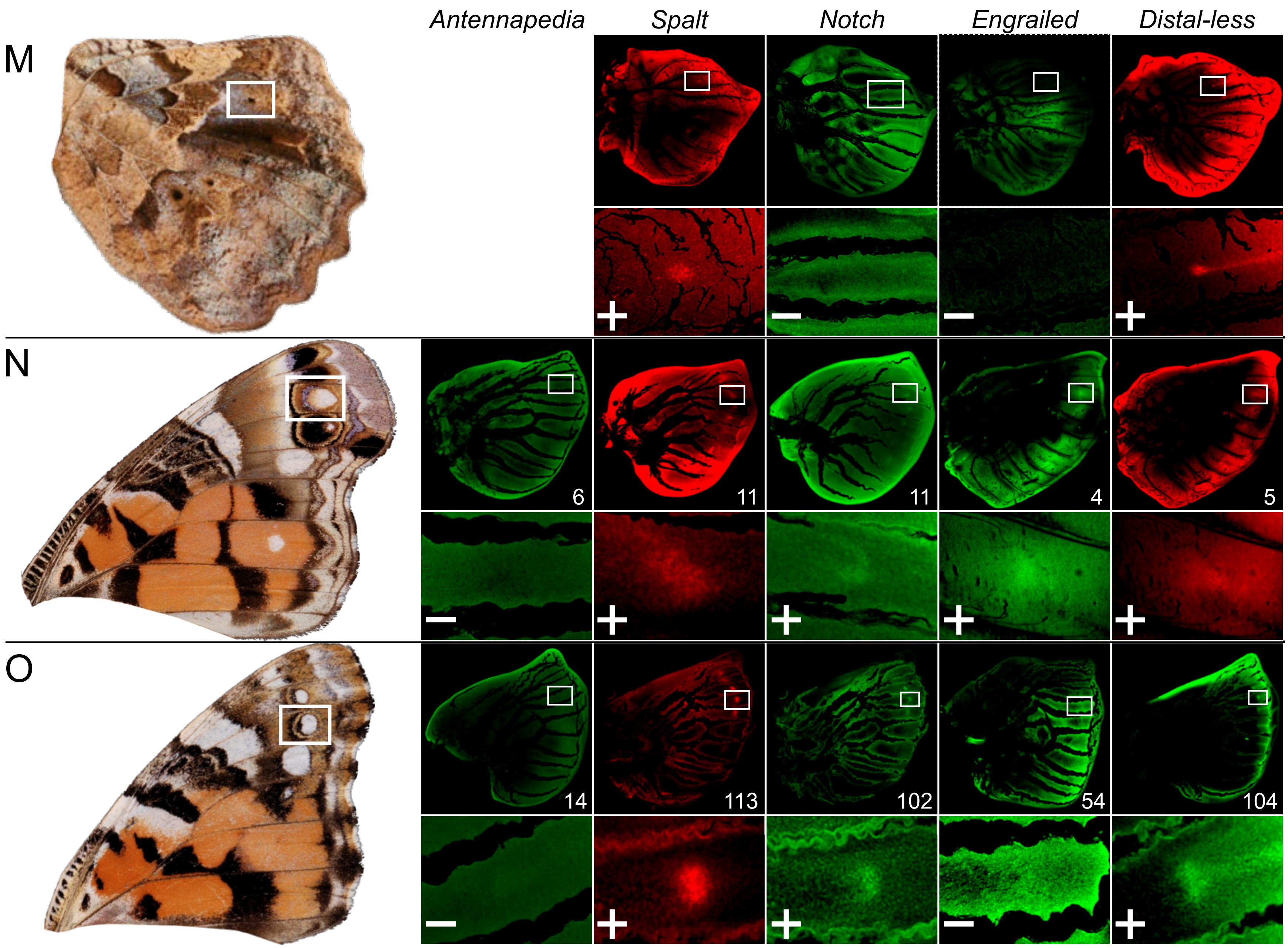

M
N
O
Antennapedia
Spalt
Notch
Engrailed
Distal-less
6
11
11
4
5
14
113
102
54
104

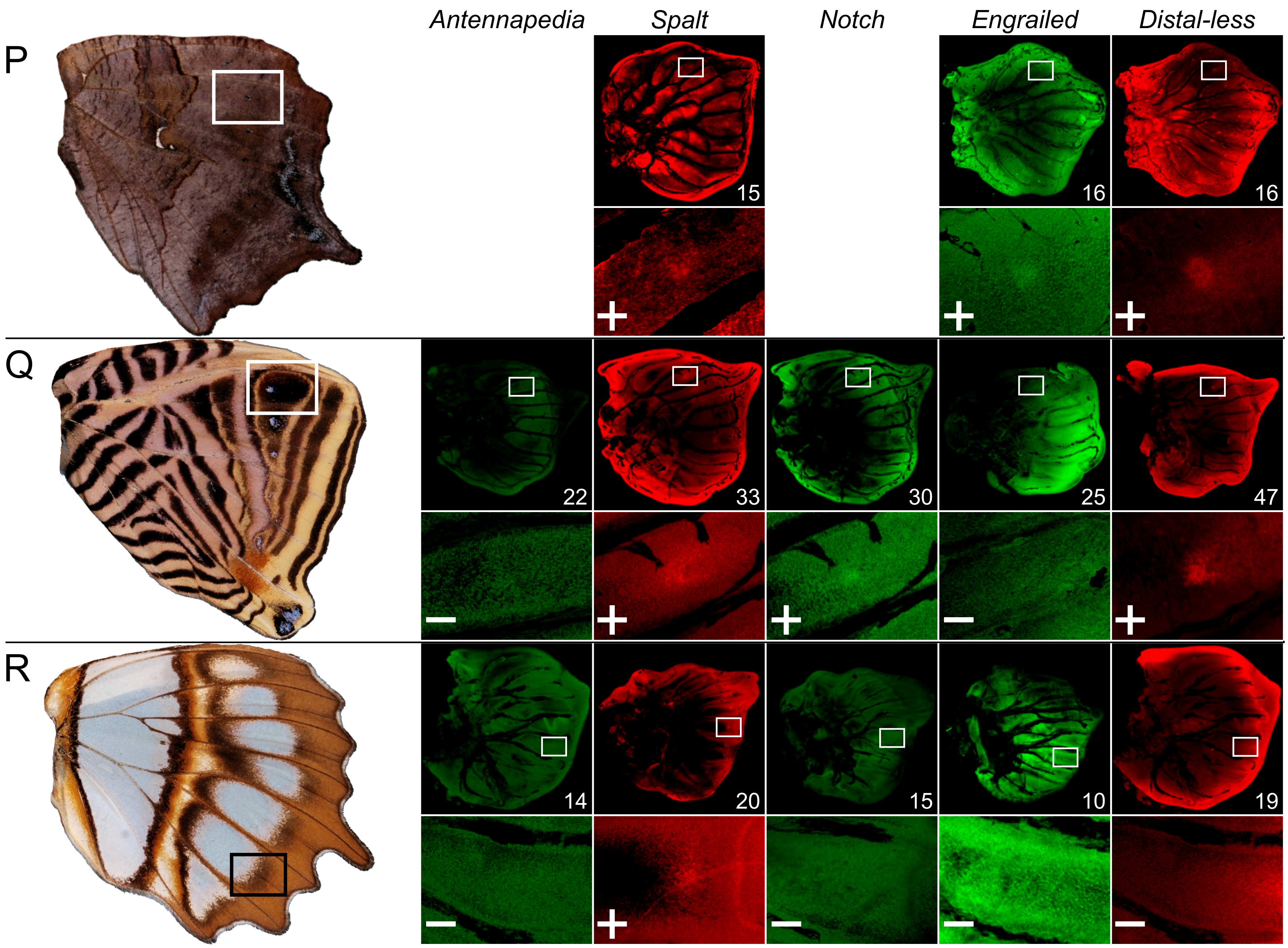
Antennapedia
Spalt
Notch
Engrailed
Distal-less
P
15
16
16
+
+
+
Q
22
33
30
25
47
−
+
+
−
+
R
14
20
15
10
19
−
+
−
−
−

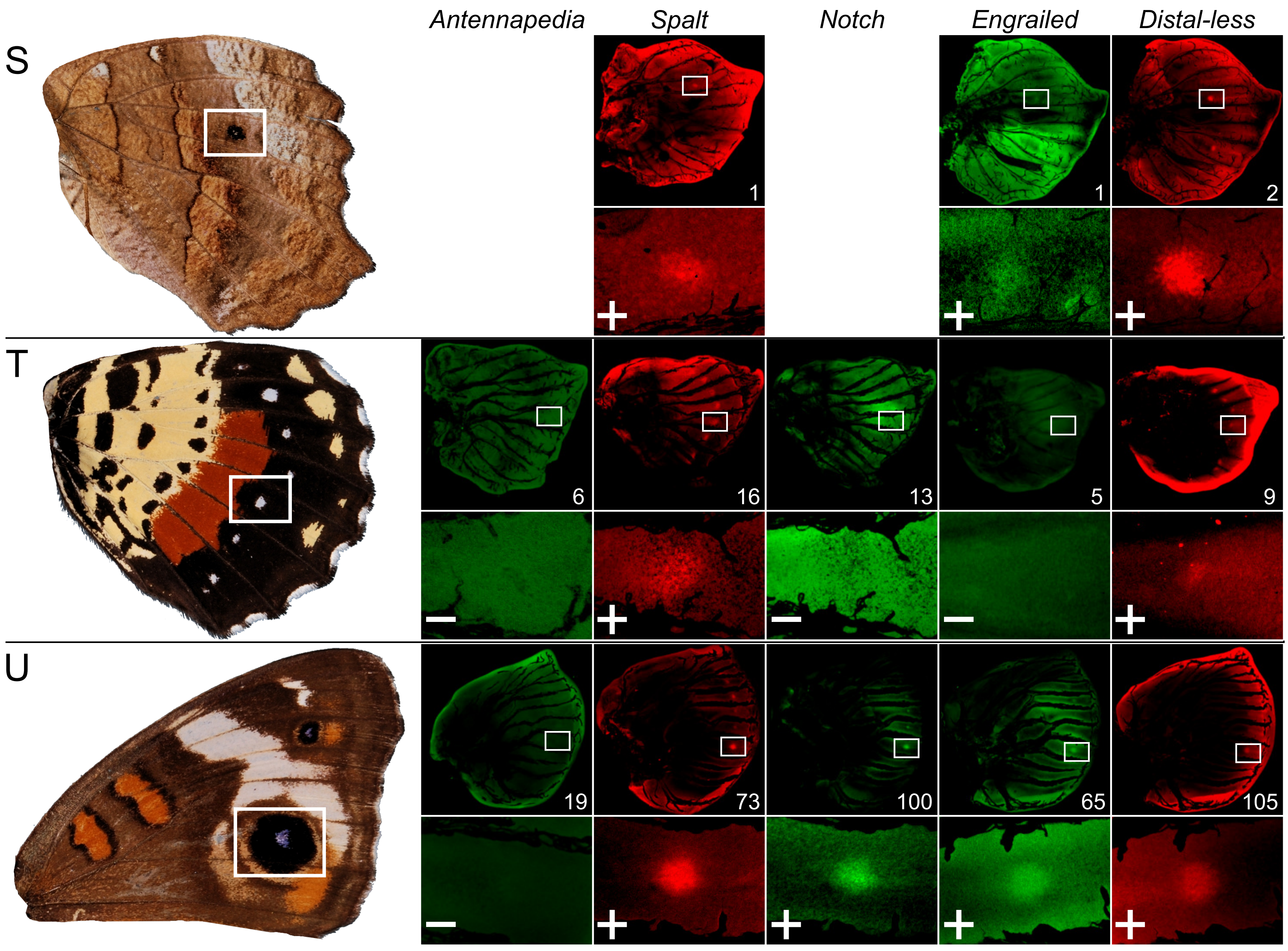
Antennapedia
Spalt
Notch
Engrailed
Distal-less
S
1
1
2
+
+
+
T
6
16
13
5
9
−
+
−
−
+
U
19
73
100
65
105
−
+
+
+
+

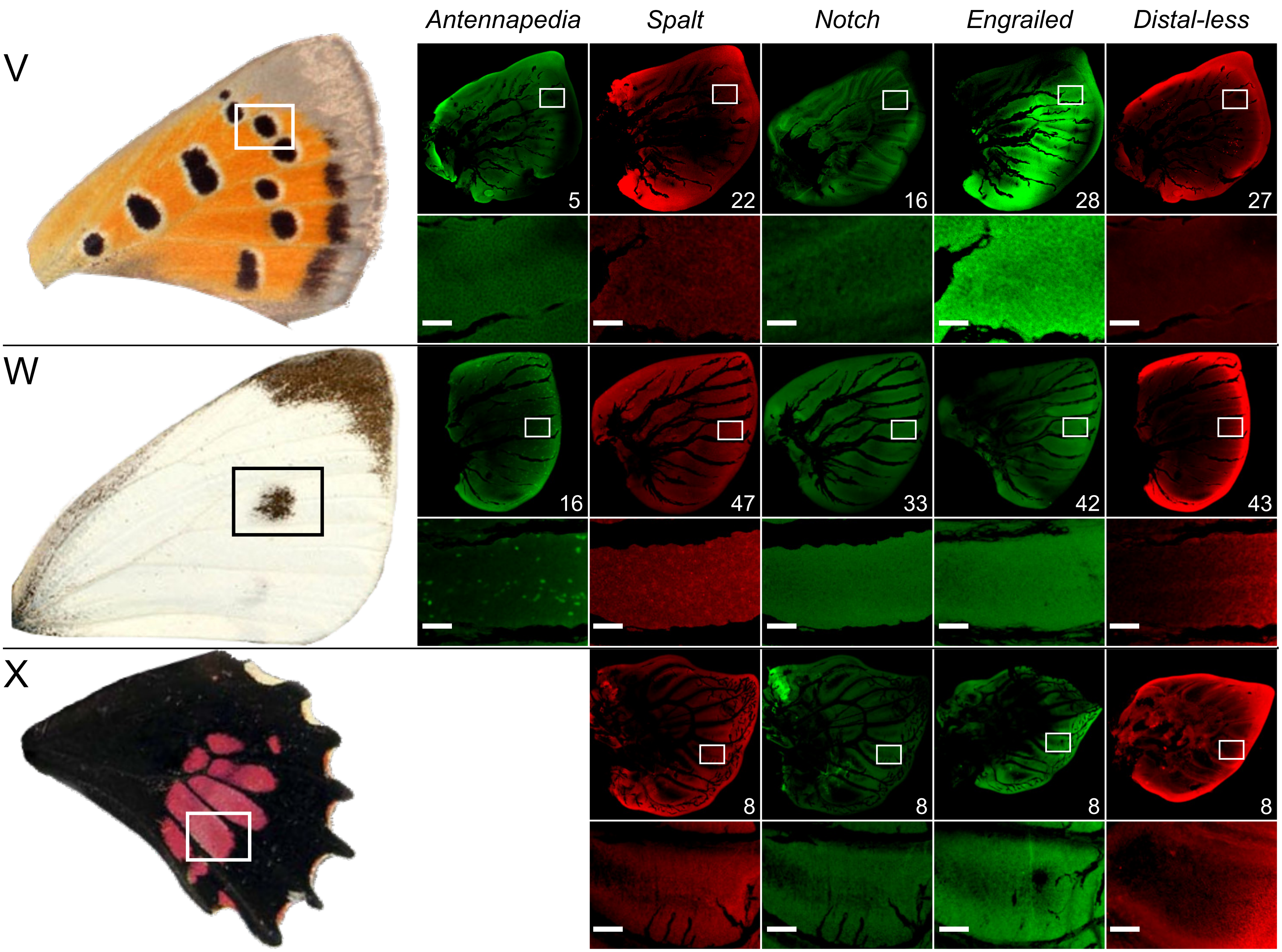

Antennapedia
Spalt
Notch
Engrailed
Distal-less
V
5
22
16
28
27
W
16
47
33
42
43
X
8
8
8
8
