# Supplementary material for: A Single Origin for Nymphalid Butterfly Eyespots Followed by Widespread Loss of Associated Gene Expression
Source: PLoS Genet. 2012 Aug 16;8(8):e1002893. doi: 10.1371/journal.pgen.1002893 (PMC3420954; doi:10.1371/journal.pgen.1002893)
Supplement: Figure S3 — Relationships among taxa used for inferring history of gene expression in future eyespot centers. Divergence times (in millions of years) within Nymphalidae from [13] and divergence times among families from [14]. Numbered nodes were used for fixing ancestral states in likelihood ratio tests (see Table S3). Expression for each gene indicated as: (−) no central expression, (+) central expression; taxon/gene combinations missing symbols indicate data not available. Asterisks (*) indicate species for which expression data are from [7], [8]. (PDF) [file pgen.1002893.s003.pdf]

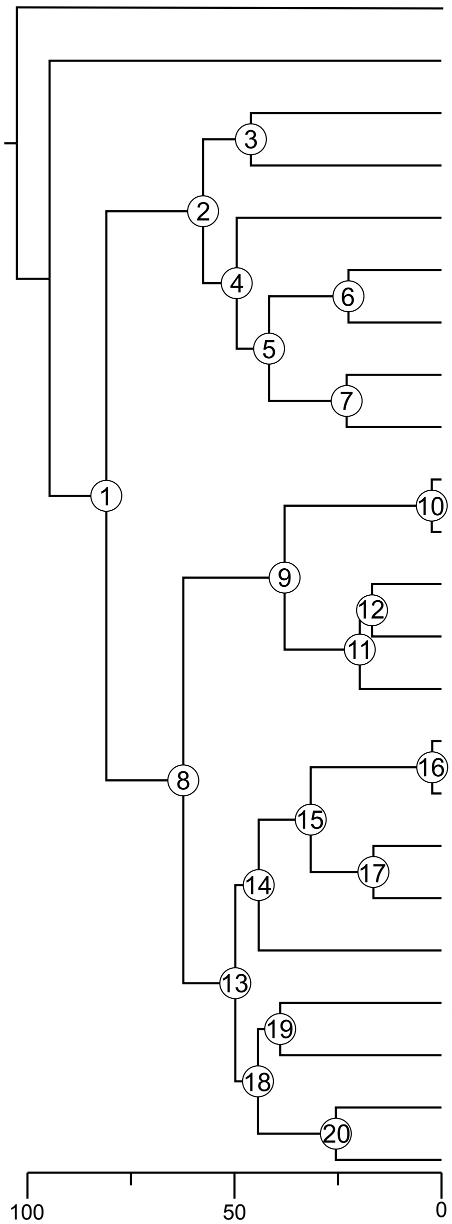

	Antp	*sal*	*Notch*	*en*	*Dll*
*Parnassius apollo**	−	−	−		−
Lycaena phlaeas	−	−	−	−	−
Caligo memnon	+	+	−	−	−
Morpho peleides	+	+		+	+
*Melanargia galathea**	+	−	+		+
*Pararge aegeria**	+	+	+		+
*Lasiommata megera**	+	+	+		+
*Heteropsis iboina**	+	+	+		+
Bicyclus anynana	+	+	+	+	+
Hamadryas amphinome	+	+	+		+
Hamadryas februa		+		−	+
Catonephele numilia		+	−	−	+
Nessaea aglaura	−	+		−	+
Myscelia cyaniris		+	−	−	+
Vanessa virginiensis	−	+	+	+	+
Vanessa cardui	−	+	+	−	+
Polygonia interrogationis		+		+	+
*Inachis io**	−	+	+		+
Colobura dirce	−	+	+	−	+
Anartia fatima		+		+	+
Junonia coenia	−	+	+	+	+
*Melitaea cinxia**	−	−	−		+
Chlosyne janais	−	+	−	−	+

Figure S3. Relationships among taxa used for inferring history of gene expression in future eyespot centers. Divergence times (in millions of years) within Nymphalidae from [13] and divergence times among families from [14]. Numbers at nodes indicate nodes used for fixing ancestral states in likelihood ratio tests (see Table S3). Expression for each gene indicated as: (−) no central expression, (+) central expression; taxon/gene combinations missing symbols indicate data not available. Asterisks (*) indicate species for which expression data are from [7,8].
