# Supplementary material for: A Single Origin for Nymphalid Butterfly Eyespots Followed by Widespread Loss of Associated Gene Expression
Source: PLoS Genet. 2012 Aug 16;8(8):e1002893. doi: 10.1371/journal.pgen.1002893 (PMC3420954; doi:10.1371/journal.pgen.1002893)
Supplement: Figure S4 — Temporal dynamics of gene expression in future eyespot centers of four nymphalid species. Graphs show the relationship between wing developmental stage and eyespot central expression (present vs. absent). Lines show best-fit logistic curves for each gene and sizes of points indicate relative number of samples observed at each developmental stage. See Figure S6 for central expression category examples. (PDF) [file pgen.1002893.s004.pdf]

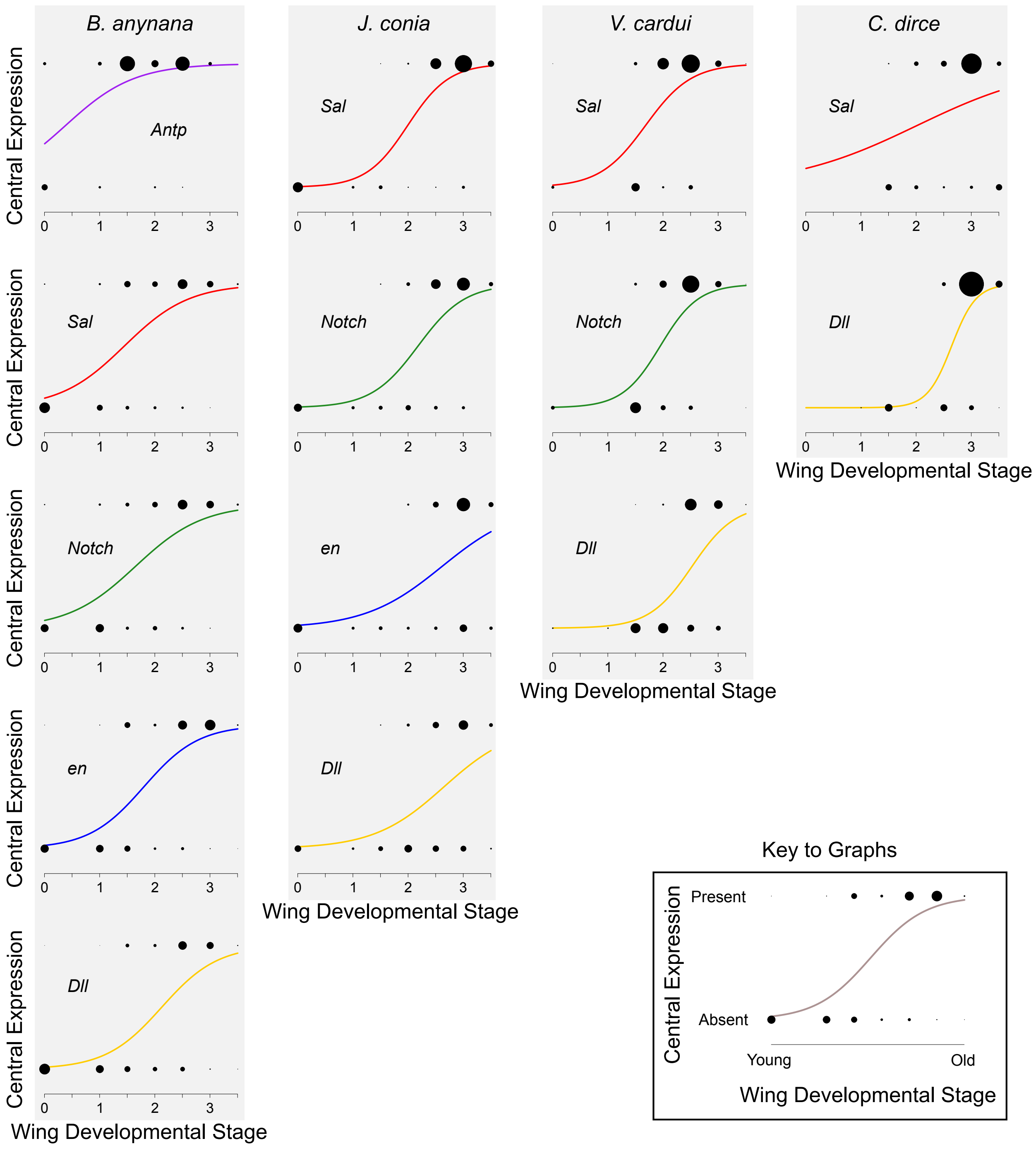

Figure S5. Temporal dynamics of gene expression in future eyespot centers of four nymphalid species. Graphs show the relationship between wing developmental stage and eyespot central expression (present vs. absent). Lines show best-fit logistic curves for each gene and sizes of points indicate relative number of samples observed at each developmental stage. See Figure S6 for central expression category examples.
