# Supplementary material for: A Single Origin for Nymphalid Butterfly Eyespots Followed by Widespread Loss of Associated Gene Expression
Source: PLoS Genet. 2012 Aug 16;8(8):e1002893. doi: 10.1371/journal.pgen.1002893 (PMC3420954; doi:10.1371/journal.pgen.1002893)

Figure S4. Co-stains showing temporal differences in expression of pairs of genes in the eyespot centers. (A-C) *Antp* before *sal*; (D-E) *Antp* before *dll*; (F-I) *sal* before *Notch*; (J) *sal* before *en*; (K-L) *sal* before *Dll*; (M-N) *Notch* before *Dll*; and (O-P) *en* before *Dll*.

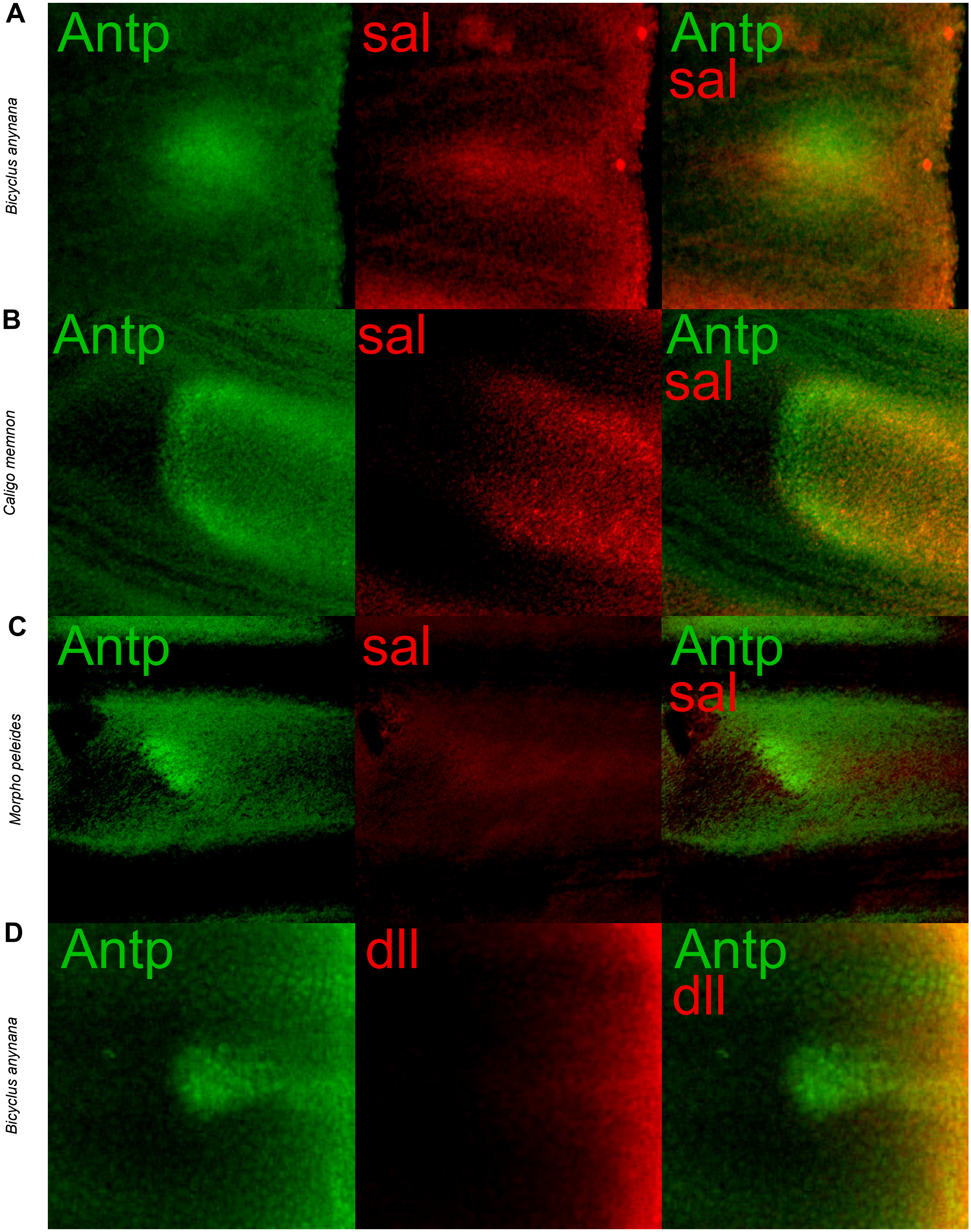
A
Antp
sal
Antp
sal
Bicyclus anynana
B
Antp
sal
Antp
sal
Caligo memnon
C
Antp
sal
Antp
sal
Morpho peleides
D
Antp
dll
Antp
dll
Bicyclus anynana

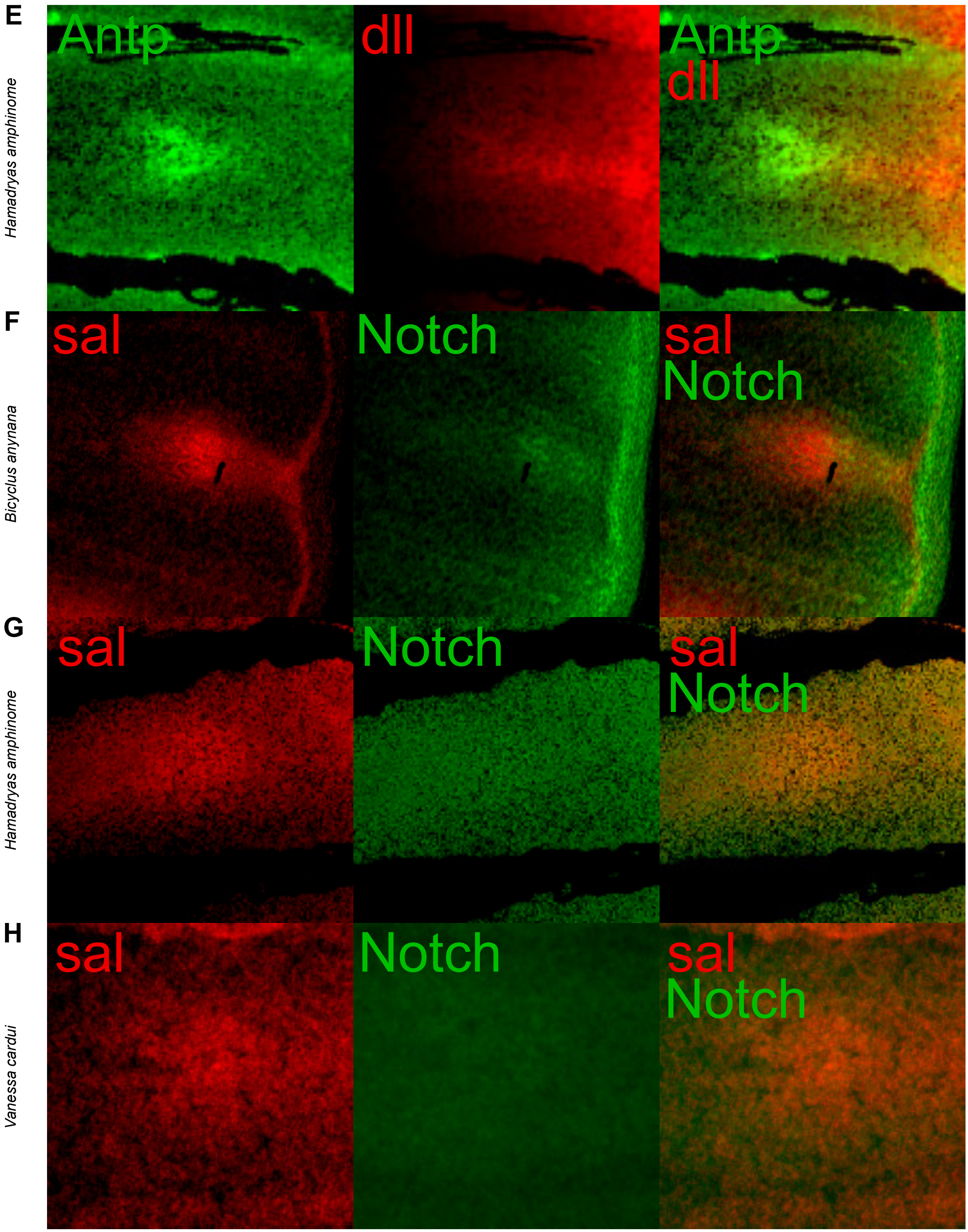
E
Hamadryas amphinome
Antp
dll
Antp
dll
F
Bicyclus anynana
sal
Notch
sal
Notch
G
Hamadryas amphinome
sal
Notch
sal
Notch
H
Vanessa cardui
sal
Notch
sal
Notch

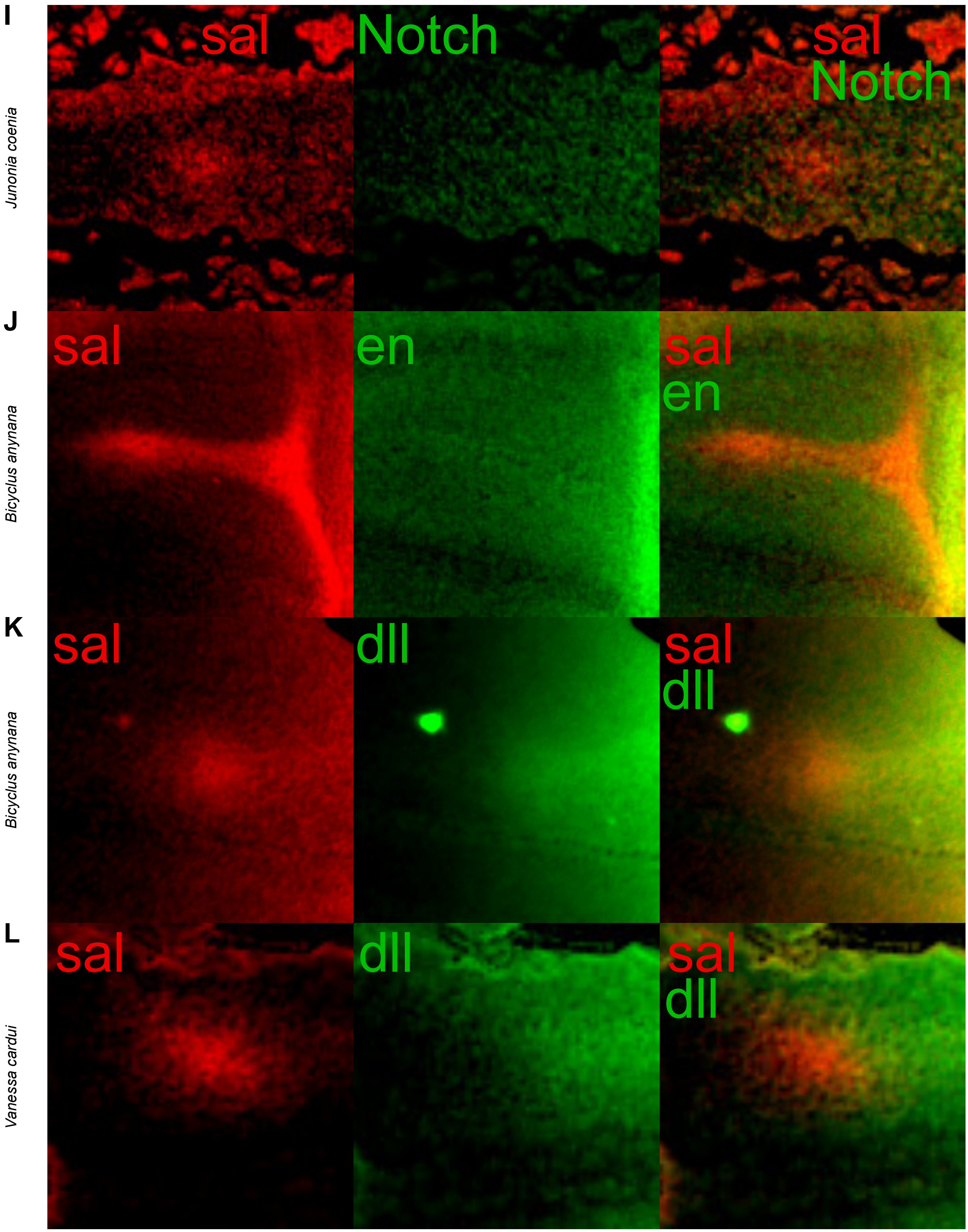
I
sal
Notch
sal
Notch
Junonia coenia
J
sal
en
sal
en
Bicyclus anynana
K
sal
dll
sal
dll
Bicyclus anynana
L
sal
dll
sal
dll
Vanessa cardui

M *Bicyclus anynana*

N *Vanessa cardui*

O *Bicyclus anynana*

P *Vanessa virginiensis*

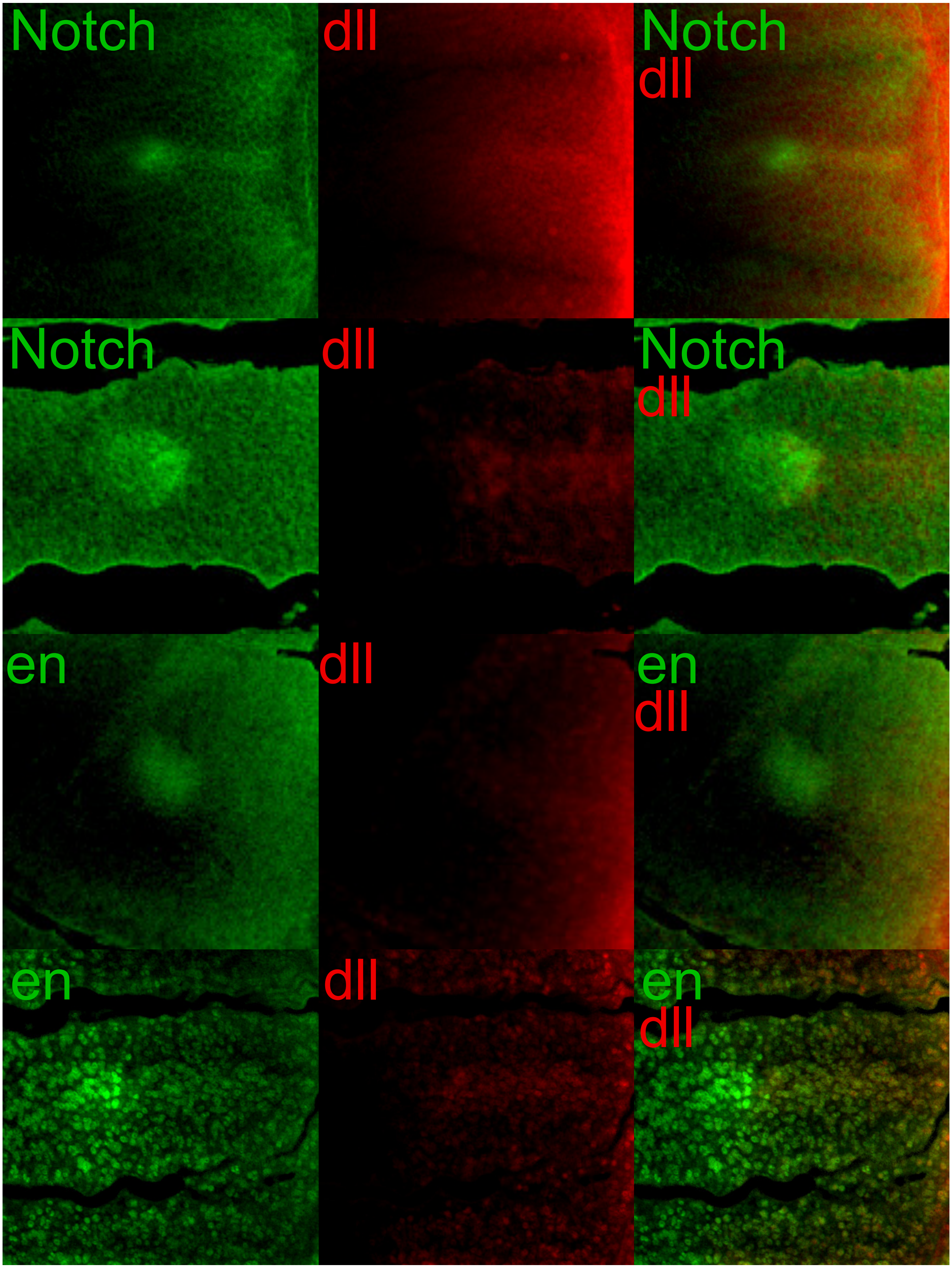

Supplement: Figure S5 — Co-stains showing temporal differences in expression of pairs of genes in the eyespot centers. (A–C) Antp before sal; (D–E) Antp before dll; (F–I) sal before Notch; (J) sal before en; (K–L) sal before Dll; (M–N) Notch before Dll; and (O–P) en before Dll. (PDF) [file pgen.1002893.s005.pdf]
