# Supplementary material for: A Single Origin for Nymphalid Butterfly Eyespots Followed by Widespread Loss of Associated Gene Expression
Source: PLoS Genet. 2012 Aug 16;8(8):e1002893. doi: 10.1371/journal.pgen.1002893 (PMC3420954; doi:10.1371/journal.pgen.1002893)
Supplement: Figure S6 — Eyespot central expression categories for temporal expression analyses. Images show anti-Sal antibody stains in the Cu1 compartment of B. anynana hindwings. Gene expression was categorized as zero (central expression absent) when no pattern of up-regulation was evident (A) or when expression was only detected in the midvein area (B). Gene expression was categorized as one (central expression present), when a clear cluster of central cells was detected, either in the presence (C) or absence (D) of expression in the midvein area. (PDF) [file pgen.1002893.s006.pdf]

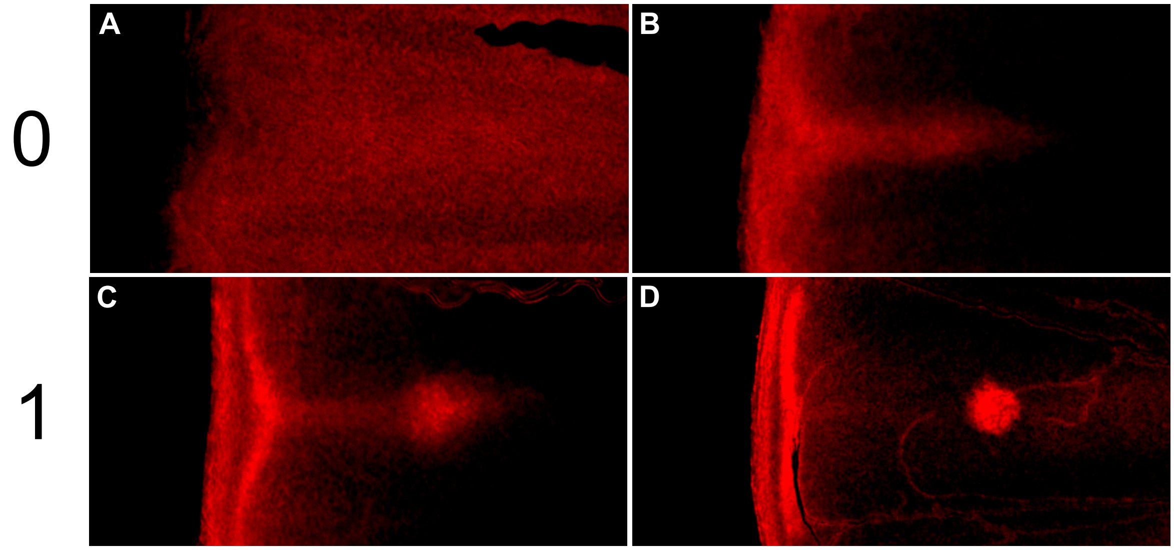

Figure S6. Focal expression categories for temporal expression analyses. Images show anti-*Sal* antibody stains in the Cu_1 compartment of *B. anynana* hindwings. Gene expression was categorized as zero (central expression absent) when no pattern of up-regulation was evident (A) or when expression was only detected in the midvein area (B). Gene expression was categorized as one (central expression present), when a clear cluster of central cells was detected, either in the presence (C) or absence (D) of expression in the midvein area.
