# Supplementary material for: A Single Origin for Nymphalid Butterfly Eyespots Followed by Widespread Loss of Associated Gene Expression
Source: PLoS Genet. 2012 Aug 16;8(8):e1002893. doi: 10.1371/journal.pgen.1002893 (PMC3420954; doi:10.1371/journal.pgen.1002893)
Supplement: Table S1 — Data matrix for presence or absence of eyespots in 399 nymphalid taxa and 17 outgroup species. (DOC) [file pgen.1002893.s007.doc]

**Table S1.** Data matrix for presence or absence of eyespots in 399 nymphalid taxa and 17 outgroup species.

| **Species** | **Eyespots** | **Family** | **Subfamily** |
| --- | --- | --- | --- |
| *Apatura iris* | Present | Nymphalidae | Apaturinae |
| *Apaturopsis cleochares* | Present | Nymphalidae | Apaturinae |
| *Asterocampa idyja* | Present | Nymphalidae | Apaturinae |
| *Chitoria ulupi* | Present | Nymphalidae | Apaturinae |
| *Doxocopa laure* | Absent | Nymphalidae | Apaturinae |
| *Eulaceura osteria* | Present | Nymphalidae | Apaturinae |
| *Euripus nyctelius* | Absent | Nymphalidae | Apaturinae |
| *Hestina mena* | Present | Nymphalidae | Apaturinae |
| *Hestinalis divona* | Absent | Nymphalidae | Apaturinae |
| *Mimathyma schrenckii* | Absent | Nymphalidae | Apaturinae |
| *Sephisa dichroa* | Present | Nymphalidae | Apaturinae |
| *Timelaea albescens* | Present | Nymphalidae | Apaturinae |
| *Archimestra teleboas* | Present | Nymphalidae | Biblidinae |
| *Ariadne enotrea* | Absent | Nymphalidae | Biblidinae |
| *Batesia hypochlora* | Absent | Nymphalidae | Biblidinae |
| *Biblis hyperia* | Absent | Nymphalidae | Biblidinae |
| *Byblia anvatara* | Absent | Nymphalidae | Biblidinae |
| *Callicore tolima* | Present | Nymphalidae | Biblidinae |
| *Catonephele numilia* | Present | Nymphalidae | Biblidinae |
| *Diaethria clymena* | Present | Nymphalidae | Biblidinae |
| *Dynamine serina* | Present | Nymphalidae | Biblidinae |
| *Ectima thecla* | Present | Nymphalidae | Biblidinae |
| *Epiphile oreas* | Present | Nymphalidae | Biblidinae |
| *Eunica viola* | Present | Nymphalidae | Biblidinae |
| *Eurytela dryope* | Present | Nymphalidae | Biblidinae |
| *Haematera pyrame* | Present | Nymphalidae | Biblidinae |
| *Hamadryas februa* | Present | Nymphalidae | Biblidinae |
| *Laringa* sp. | Absent | Nymphalidae | Biblidinae |
| *Lucinia cadma* | Present | Nymphalidae | Biblidinae |
| *Mesoxantha ethosea* | Absent | Nymphalidae | Biblidinae |
| *Mestra hypermestra* | Absent | Nymphalidae | Biblidinae |
| *Myscelia capenas* | Present | Nymphalidae | Biblidinae |
| *Neptidopsis ophione* | Present | Nymphalidae | Biblidinae |
| *Nessaea aglaura* | Present | Nymphalidae | Biblidinae |
| *Nica flavilla* | Present | Nymphalidae | Biblidinae |
| *Panacea regina* | Present | Nymphalidae | Biblidinae |
| *Pyrrhogyra crameri* | Absent | Nymphalidae | Biblidinae |
| *Sevenia boisduvali* | Present | Nymphalidae | Biblidinae |
| *Temenis laothoe* | Present | Nymphalidae | Biblidinae |
| *Vila azeka* | Absent | Nymphalidae | Biblidinae |
| *Calinaga buddha* | Absent | Nymphalidae | Calinaginae |
| *Agatasa calydonia* | Absent | Nymphalidae | Charaxinae |
| *Agrias* sp. | Present | Nymphalidae | Charaxinae |
| *Anaea troglodyta* | Absent | Nymphalidae | Charaxinae |
| *Anaeomorpha splendida* | Present | Nymphalidae | Charaxinae |
| *Archaeoprepona demophon* | Present | Nymphalidae | Charaxinae |
| *Charaxes castor* | Present | Nymphalidae | Charaxinae |
| *Coenophlebia* sp. | Absent | Nymphalidae | Charaxinae |
| *Consul fabius* | Absent | Nymphalidae | Charaxinae |
| *Euxanthe eurinome* | Absent | Nymphalidae | Charaxinae |
| *Fountainea ryphea* | Absent | Nymphalidae | Charaxinae |
| *Hypna clytemnestra* | Absent | Nymphalidae | Charaxinae |
| *Memphis appias* | Present | Nymphalidae | Charaxinae |
| *Palla decius* | Present | Nymphalidae | Charaxinae |
| *Polygrapha tyrianthina* | Absent | Nymphalidae | Charaxinae |
| *Polyura maeri* | Present | Nymphalidae | Charaxinae |
| *Prepona* sp. | Present | Nymphalidae | Charaxinae |
| *Prothoe franck* | Present | Nymphalidae | Charaxinae |
| *Siderone marthesia* | Absent | Nymphalidae | Charaxinae |
| *Zaretis* sp. | Present | Nymphalidae | Charaxinae |
| *Chersonesia rahria* | Present | Nymphalidae | Cyrestinae |
| *Cyrestis thyodamas* | Present | Nymphalidae | Cyrestinae |
| *Marpesia eleuchea* | Present | Nymphalidae | Cyrestinae |
| *Aeria eurimedea* | Absent | Nymphalidae | Danainae |
| *Amauris ellioti* | Absent | Nymphalidae | Danainae |
| *Anetia briarea* | Present | Nymphalidae | Danainae |
| *Athesis clearista* | Absent | Nymphalidae | Danainae |
| *Athyrtis mechanitis* | Absent | Nymphalidae | Danainae |
| *Brevioleria aelia* | Absent | Nymphalidae | Danainae |
| *Callithomia lenea* | Absent | Nymphalidae | Danainae |
| *Ceratinia neso* | Absent | Nymphalidae | Danainae |
| *Danaus plexippus* | Absent | Nymphalidae | Danainae |
| *Dircenna dero* | Absent | Nymphalidae | Danainae |
| *Elzunia pavoni* | Absent | Nymphalidae | Danainae |
| *Episcada apuleia* | Absent | Nymphalidae | Danainae |
| *Epityches eupompe* | Absent | Nymphalidae | Danainae |
| *Euploea camaralzeman* | Absent | Nymphalidae | Danainae |
| *Forbestra equicola* | Absent | Nymphalidae | Danainae |
| *Godyris duillia* | Absent | Nymphalidae | Danainae |
| *Greta oto* | Absent | Nymphalidae | Danainae |
| *Heterosais guilia* | Absent | Nymphalidae | Danainae |
| *Hyalenna* sp. | Absent | Nymphalidae | Danainae |
| *Hyaliris antea* | Absent | Nymphalidae | Danainae |
| *Hypoleria lavinia* | Absent | Nymphalidae | Danainae |
| *Hyposcada anchialia* | Absent | Nymphalidae | Danainae |
| *Hypothyris daphnis* | Absent | Nymphalidae | Danainae |
| *Idea leucone* | Absent | Nymphalidae | Danainae |
| *Ideopsis juventa* | Absent | Nymphalidae | Danainae |
| *Ithomia drymo* | Absent | Nymphalidae | Danainae |
| *Ituna ilione* | Absent | Nymphalidae | Danainae |
| *Lycorea halia* | Absent | Nymphalidae | Danainae |
| *Mcclungia cymo* | Absent | Nymphalidae | Danainae |
| *Mechanitis polymnia* | Absent | Nymphalidae | Danainae |
| *Megoleria orestilla* | Absent | Nymphalidae | Danainae |
| *Melinaea menophilus* | Absent | Nymphalidae | Danainae |
| *Methona themisto* | Absent | Nymphalidae | Danainae |
| *Napeogenes pharo* | Absent | Nymphalidae | Danainae |
| *Oleria onega* | Absent | Nymphalidae | Danainae |
| *Ollantaya aeginata* | Absent | Nymphalidae | Danainae |
| *Olyras insignis* | Absent | Nymphalidae | Danainae |
| *Pagyris cymothoe* | Absent | Nymphalidae | Danainae |
| *Paititia neglecta* | Absent | Nymphalidae | Danainae |
| *Parantica aspasia* | Absent | Nymphalidae | Danainae |
| *Patricia dercillidas* | Absent | Nymphalidae | Danainae |
| *Placidina euryanassa* | Absent | Nymphalidae | Danainae |
| *Pseudoscada timna* | Absent | Nymphalidae | Danainae |
| *Pteronymia teresita* | Absent | Nymphalidae | Danainae |
| *Sais rosalia* | Absent | Nymphalidae | Danainae |
| *Scada reckia* | Absent | Nymphalidae | Danainae |
| *Tellervo zoilus* | Absent | Nymphalidae | Danainae |
| *Thyridia psidii* | Absent | Nymphalidae | Danainae |
| *Tirumala limniacea* | Absent | Nymphalidae | Danainae |
| *Tithorea harmonia* | Absent | Nymphalidae | Danainae |
| *Veladyris pardalis* | Absent | Nymphalidae | Danainae |
| *Velamysta pupilla* | Absent | Nymphalidae | Danainae |
| *Acraea pseudegina* | Absent | Nymphalidae | Heliconiinae |
| *Actinote surima* | Absent | Nymphalidae | Heliconiinae |
| *Agraulis vanillae* | Present | Nymphalidae | Heliconiinae |
| *Algia fasciata* | Present | Nymphalidae | Heliconiinae |
| *Algiachroa woodfordi* | Present | Nymphalidae | Heliconiinae |
| *Altinote stratonice* | Absent | Nymphalidae | Heliconiinae |
| *Argynnis paphia* | Present | Nymphalidae | Heliconiinae |
| *Bematistes alcinoe* | Absent | Nymphalidae | Heliconiinae |
| *Boloria napaea* | Present | Nymphalidae | Heliconiinae |
| *Brenthis ino* | Present | Nymphalidae | Heliconiinae |
| *Cethosia cyane* | Present | Nymphalidae | Heliconiinae |
| *Cirrhochroa tyche* | Present | Nymphalidae | Heliconiinae |
| *Cupha prosope* | Present | Nymphalidae | Heliconiinae |
| *Dione glycera* | Absent | Nymphalidae | Heliconiinae |
| *Dryadula phaetusa* | Absent | Nymphalidae | Heliconiinae |
| *Dryas iulia* | Absent | Nymphalidae | Heliconiinae |
| *Eueides isabella* | Absent | Nymphalidae | Heliconiinae |
| *Euptoieta claudia* | Present | Nymphalidae | Heliconiinae |
| *Heliconius hecale* | Absent | Nymphalidae | Heliconiinae |
| *Issoria eugenia* | Absent | Nymphalidae | Heliconiinae |
| *Lachnoptera anticlia* | Present | Nymphalidae | Heliconiinae |
| *Pardopsis punctatissima* | Absent | Nymphalidae | Heliconiinae |
| *Phalanta phalantha* | Present | Nymphalidae | Heliconiinae |
| *Philaethria wernickei* | Present | Nymphalidae | Heliconiinae |
| *Terinos atlita* | Present | Nymphalidae | Heliconiinae |
| *Vagrans egista* | Present | Nymphalidae | Heliconiinae |
| *Vindula arsinoe* | Present | Nymphalidae | Heliconiinae |
| *Yramea cytheris* | Present | Nymphalidae | Heliconiinae |
| *Libyt carinenta* | Absent | Nymphalidae | Libytheinae |
| *Libythea celtis* | Absent | Nymphalidae | Libytheinae |
| *Libytheana terena* | Absent | Nymphalidae | Libytheinae |
| *Adelpha bredowi* | Present | Nymphalidae | Limenitinae |
| *Aterica galene* | Present | Nymphalidae | Limenitinae |
| *Athyma jina* | Present | Nymphalidae | Limenitinae |
| *Bassarona dunya* | Present | Nymphalidae | Limenitinae |
| *Bebearia sophus* | Present | Nymphalidae | Limenitinae |
| *Catuna crithea* | Present | Nymphalidae | Limenitinae |
| *Crenidomimas concordia* | Present | Nymphalidae | Limenitinae |
| *Cymothoe caenis* | Absent | Nymphalidae | Limenitinae |
| *Dophla evelina* | Absent | Nymphalidae | Limenitinae |
| *Euphaedra herberti* | Absent | Nymphalidae | Limenitinae |
| *Euptera elabontas* | Present | Nymphalidae | Limenitinae |
| *Euriphene tadema* | Present | Nymphalidae | Limenitinae |
| *Euryphura chalcis* | Present | Nymphalidae | Limenitinae |
| *Hamanumida daedalus* | Present | Nymphalidae | Limenitinae |
| *Harma theobene* | Absent | Nymphalidae | Limenitinae |
| *Lasippa tiga* | Absent | Nymphalidae | Limenitinae |
| *Lebadea martha* | Absent | Nymphalidae | Limenitinae |
| *Lexias pardalis* | Absent | Nymphalidae | Limenitinae |
| *Limenitis reducta* | Absent | Nymphalidae | Limenitinae |
| *Moduza procris* | Absent | Nymphalidae | Limenitinae |
| *Neptis ida* | Absent | Nymphalidae | Limenitinae |
| *Pantoporia sandaka* | Absent | Nymphalidae | Limenitinae |
| *Parthenos sylvia* | Absent | Nymphalidae | Limenitinae |
| *Pseudacraea poggei* | Absent | Nymphalidae | Limenitinae |
| *Pseudoneptis bugandensis* | Absent | Nymphalidae | Limenitinae |
| *Tanaecia julii* | Present | Nymphalidae | Limenitinae |
| *Aglais urticae* | Absent | Nymphalidae | Nymphalinae |
| *Anartia amathea* | Absent | Nymphalidae | Nymphalinae |
| *Antanartia delius* | Present | Nymphalidae | Nymphalinae |
| *Anthanassa texana* | Present | Nymphalidae | Nymphalinae |
| *Antillea pelops* | Present | Nymphalidae | Nymphalinae |
| *Araschnia levana* | Present | Nymphalidae | Nymphalinae |
| *Atlantea pantoni* | Present | Nymphalidae | Nymphalinae |
| *Baeotus beotus* | Present | Nymphalidae | Nymphalinae |
| *Castilia castilla* | Absent | Nymphalidae | Nymphalinae |
| *Catacroptera cloanthe* | Present | Nymphalidae | Nymphalinae |
| *Chlosyne janais* | Absent | Nymphalidae | Nymphalinae |
| *Colobura dirce* | Present | Nymphalidae | Nymphalinae |
| *Dagon pusillus* | Present | Nymphalidae | Nymphalinae |
| *Doleschallia bisaltide* | Present | Nymphalidae | Nymphalinae |
| *Eresia eunice* | Absent | Nymphalidae | Nymphalinae |
| *Euphydryas phaeton* | Absent | Nymphalidae | Nymphalinae |
| *Gnathotriche exclamationis* | Absent | Nymphalidae | Nymphalinae |
| *Higginsius fasciata* | Absent | Nymphalidae | Nymphalinae |
| *Historis odius* | Present | Nymphalidae | Nymphalinae |
| *Hypanartia paullus* | Present | Nymphalidae | Nymphalinae |
| *Hypolimnas bolina* | Present | Nymphalidae | Nymphalinae |
| *Janatella leucodesma* | Present | Nymphalidae | Nymphalinae |
| *Junonia oenone* | Present | Nymphalidae | Nymphalinae |
| *Kallima paralekta* | Present | Nymphalidae | Nymphalinae |
| *Kallimoides rumia* | Present | Nymphalidae | Nymphalinae |
| *Kaniska canace* | Present | Nymphalidae | Nymphalinae |
| *Mallika jacksoni* | Present | Nymphalidae | Nymphalinae |
| *Mazia amazonica* | Present | Nymphalidae | Nymphalinae |
| *Melitaea cinxia* | Present | Nymphalidae | Nymphalinae |
| *Metamorpha elissa* | Absent | Nymphalidae | Nymphalinae |
| *Microtia elva* | Absent | Nymphalidae | Nymphalinae |
| *Mynes geoffroyi* | Absent | Nymphalidae | Nymphalinae |
| *Napeocles jucunda* | Present | Nymphalidae | Nymphalinae |
| *Nymphalis polychloros* | Present | Nymphalidae | Nymphalinae |
| *Ortilia liriope* | Present | Nymphalidae | Nymphalinae |
| *Phyciodes cocyta* | Present | Nymphalidae | Nymphalinae |
| *Phystis simois* | Present | Nymphalidae | Nymphalinae |
| *Poladryas arachne* | Present | Nymphalidae | Nymphalinae |
| *Polygonia c aureum* | Present | Nymphalidae | Nymphalinae |
| *Precis octavia* | Present | Nymphalidae | Nymphalinae |
| *Protogoniomorpha anacardii* | Present | Nymphalidae | Nymphalinae |
| *Pycina zamba* | Present | Nymphalidae | Nymphalinae |
| *Rhinopalpa polynice* | Present | Nymphalidae | Nymphalinae |
| *Salamis anteva* | Present | Nymphalidae | Nymphalinae |
| *Siproeta stelenes* | Absent | Nymphalidae | Nymphalinae |
| *Smyrna blomfildia* | Present | Nymphalidae | Nymphalinae |
| *Symbrenthia lilea* | Present | Nymphalidae | Nymphalinae |
| *Tegosa claudina* | Present | Nymphalidae | Nymphalinae |
| *Telenassa teletusa* | Present | Nymphalidae | Nymphalinae |
| *Tigridia acesta* | Absent | Nymphalidae | Nymphalinae |
| *Vanessa atalanta* | Present | Nymphalidae | Nymphalinae |
| *Vanessula milca* | Absent | Nymphalidae | Nymphalinae |
| *Yoma algina* | Present | Nymphalidae | Nymphalinae |
| *Amnosia decora* | Present | Nymphalidae | Pseudergolinae |
| *Dichorragia nesimachus* | Absent | Nymphalidae | Pseudergolinae |
| *Pseudergolis wedah* | Present | Nymphalidae | Pseudergolinae |
| *Stibochiona nicea* | Absent | Nymphalidae | Pseudergolinae |
| *Aemona lena* | Present | Nymphalidae | Satyrinae |
| *Aeropetes tulbaghia* | Present | Nymphalidae | Satyrinae |
| *Altiapa klossi* | Present | Nymphalidae | Satyrinae |
| *Amathusia phidippus* | Present | Nymphalidae | Satyrinae |
| *Amathuxidia amythaon* | Present | Nymphalidae | Satyrinae |
| *Amphidecta calliomma* | Present | Nymphalidae | Satyrinae |
| *Antirrhea philoctetes* | Present | Nymphalidae | Satyrinae |
| *Apexacuta astoreth* | Present | Nymphalidae | Satyrinae |
| *Aphantopus hyperantus* | Present | Nymphalidae | Satyrinae |
| *Archeuptychia cluena* | Present | Nymphalidae | Satyrinae |
| *Arethusana arethusa* | Present | Nymphalidae | Satyrinae |
| *Argynnina cyrila* | Present | Nymphalidae | Satyrinae |
| *Argyronympha pulchra* | Absent | Nymphalidae | Satyrinae |
| *Argyrophenga antipodium* | Present | Nymphalidae | Satyrinae |
| *Argyrophorus argenteus* | Present | Nymphalidae | Satyrinae |
| *Auca coctei* | Present | Nymphalidae | Satyrinae |
| *Berberia lambessanus* | Present | Nymphalidae | Satyrinae |
| *Bia actorion* | Present | Nymphalidae | Satyrinae |
| *Bicyclus anynana* | Present | Nymphalidae | Satyrinae |
| *Brassolis sophorae* | Present | Nymphalidae | Satyrinae |
| *Brintesia circe* | Present | Nymphalidae | Satyrinae |
| *Caerois* sp. | Absent | Nymphalidae | Satyrinae |
| *Caeruleuptychia lobelia* | Present | Nymphalidae | Satyrinae |
| *Caligo telamonius* | Present | Nymphalidae | Satyrinae |
| *Calisto obscura* | Present | Nymphalidae | Satyrinae |
| *Cassionympha cassius* | Present | Nymphalidae | Satyrinae |
| *Catoblepia orgetorix* | Present | Nymphalidae | Satyrinae |
| *Cepheuptychia* sp. | Present | Nymphalidae | Satyrinae |
| *Cercyonis pegala* | Present | Nymphalidae | Satyrinae |
| *Chazara briseis* | Present | Nymphalidae | Satyrinae |
| *Chillanella stelligera* | Present | Nymphalidae | Satyrinae |
| *Chloreuptychia catarina* | Present | Nymphalidae | Satyrinae |
| *Chonala miyafagi* | Present | Nymphalidae | Satyrinae |
| *Cissia proba* | Present | Nymphalidae | Satyrinae |
| *Cithaerias pireta* | Present | Nymphalidae | Satyrinae |
| *Coenonympha pamphilus* | Present | Nymphalidae | Satyrinae |
| *Corades cistene* | Present | Nymphalidae | Satyrinae |
| *Cosmosatyrus leptoneuroides* | Present | Nymphalidae | Satyrinae |
| *Cyllogenes woolleti* | Present | Nymphalidae | Satyrinae |
| *Cyllopsis pertepida* | Present | Nymphalidae | Satyrinae |
| *Daedalma* sp. | Present | Nymphalidae | Satyrinae |
| *Dasyopthalma rusina* | Present | Nymphalidae | Satyrinae |
| *Dira clytus* | Present | Nymphalidae | Satyrinae |
| *Discophora necho* | Present | Nymphalidae | Satyrinae |
| *Dodonidia helmsi* | Present | Nymphalidae | Satyrinae |
| *Dynastor darius* | Present | Nymphalidae | Satyrinae |
| *Elina montrolii* | Present | Nymphalidae | Satyrinae |
| *Elymnias casiphone* | Absent | Nymphalidae | Satyrinae |
| *Enodia portlandia* | Present | Nymphalidae | Satyrinae |
| *Erebia oeme* | Present | Nymphalidae | Satyrinae |
| *Erebiola butleri* | Present | Nymphalidae | Satyrinae |
| *Eretris* sp. | Present | Nymphalidae | Satyrinae |
| *Erichthodes antonina* | Present | Nymphalidae | Satyrinae |
| *Erites argentina* | Present | Nymphalidae | Satyrinae |
| *Etcheverrius chiliensis* | Absent | Nymphalidae | Satyrinae |
| *Eteona tisiphone* | Absent | Nymphalidae | Satyrinae |
| *Ethope noirei* | Present | Nymphalidae | Satyrinae |
| *Euptychia* sp. | Present | Nymphalidae | Satyrinae |
| *Euptychoides castrensis* | Present | Nymphalidae | Satyrinae |
| *Faunis menado* | Present | Nymphalidae | Satyrinae |
| *Faunula leucoglene* | Present | Nymphalidae | Satyrinae |
| *Foetterleia schreineri* | Present | Nymphalidae | Satyrinae |
| *Forsterinaria quantius* | Present | Nymphalidae | Satyrinae |
| *Geitoneura klugii* | Present | Nymphalidae | Satyrinae |
| *Gnophodes chelys* | Present | Nymphalidae | Satyrinae |
| *Godartiana muscosa* | Present | Nymphalidae | Satyrinae |
| *Guaianaza pronophila* | Present | Nymphalidae | Satyrinae |
| *Haetera piera* | Present | Nymphalidae | Satyrinae |
| *Hallelesis halyma* | Present | Nymphalidae | Satyrinae |
| *Harjesia blanda* | Present | Nymphalidae | Satyrinae |
| *Harsiesis hygea* | Present | Nymphalidae | Satyrinae |
| *Henotesia simonsii* | Present | Nymphalidae | Satyrinae |
| *Hermeuptychia hermes* | Present | Nymphalidae | Satyrinae |
| *Heteronympha merope* | Present | Nymphalidae | Satyrinae |
| *Hipparchia semele* | Present | Nymphalidae | Satyrinae |
| *Hyantis hodeva* | Present | Nymphalidae | Satyrinae |
| *Hypocysta adiante* | Present | Nymphalidae | Satyrinae |
| *Hyponephele cadusia* | Present | Nymphalidae | Satyrinae |
| *Ianussiusa maso* | Present | Nymphalidae | Satyrinae |
| *Idioneurula eremita* | Present | Nymphalidae | Satyrinae |
| *Karanasa pamira* | Present | Nymphalidae | Satyrinae |
| *Kirinia roxelana* | Present | Nymphalidae | Satyrinae |
| *Lamprolenis nitida* | Present | Nymphalidae | Satyrinae |
| *Lasiommata megera* | Present | Nymphalidae | Satyrinae |
| *Lasiophila cirta* | Present | Nymphalidae | Satyrinae |
| *Lethe minerva* | Present | Nymphalidae | Satyrinae |
| *Lopinga achine* | Present | Nymphalidae | Satyrinae |
| *Lyela myops* | Present | Nymphalidae | Satyrinae |
| *Lymanopoda rana* | Present | Nymphalidae | Satyrinae |
| *Magneuptychia* sp. | Present | Nymphalidae | Satyrinae |
| *Manataria hercyna* | Present | Nymphalidae | Satyrinae |
| *Manerebia cyclopina* | Present | Nymphalidae | Satyrinae |
| *Maniola jurtina* | Present | Nymphalidae | Satyrinae |
| *Megisto* sp. | Present | Nymphalidae | Satyrinae |
| *Melanargia galathea* | Present | Nymphalidae | Satyrinae |
| *Melanitis leda* | Present | Nymphalidae | Satyrinae |
| *Moneuptychia paeon* | Present | Nymphalidae | Satyrinae |
| *Morpho helenor* | Present | Nymphalidae | Satyrinae |
| *Morphotenaris schoenbergi* | Present | Nymphalidae | Satyrinae |
| *Mycalesis terminus* | Present | Nymphalidae | Satyrinae |
| *Mygona irmina* | Present | Nymphalidae | Satyrinae |
| *Narope* sp. | Present | Nymphalidae | Satyrinae |
| *Nelia nemyroides* | Absent | Nymphalidae | Satyrinae |
| *Neocoenyra petersi* | Present | Nymphalidae | Satyrinae |
| *Neominois ridingsii* | Present | Nymphalidae | Satyrinae |
| *Neonympha areolata* | Present | Nymphalidae | Satyrinae |
| *Neope bremeri* | Present | Nymphalidae | Satyrinae |
| *Neorina* sp. | Present | Nymphalidae | Satyrinae |
| *Nesoxenica leprea* | Present | Nymphalidae | Satyrinae |
| *Oeneis jutta* | Present | Nymphalidae | Satyrinae |
| *Opoptera syme* | Present | Nymphalidae | Satyrinae |
| *Opsiphanes quiteria* | Present | Nymphalidae | Satyrinae |
| *Oreixenica lathoniella* | Present | Nymphalidae | Satyrinae |
| *Oressinoma typhla* | Absent | Nymphalidae | Satyrinae |
| *Orsotriaena medus* | Present | Nymphalidae | Satyrinae |
| *Oxeoschistus leucospilos* | Present | Nymphalidae | Satyrinae |
| *Palaeonympha opalina* | Present | Nymphalidae | Satyrinae |
| *Pampasatyrus gyrtone* | Present | Nymphalidae | Satyrinae |
| *Panyapedaliodes drymaea* | Absent | Nymphalidae | Satyrinae |
| *Paralasa jordana* | Present | Nymphalidae | Satyrinae |
| *Paralethe dendrophilus* | Present | Nymphalidae | Satyrinae |
| *Parapedaliodes parepa* | Present | Nymphalidae | Satyrinae |
| *Pararge aegeria* | Present | Nymphalidae | Satyrinae |
| *Parataygetis albinonata* | Present | Nymphalidae | Satyrinae |
| *Pareuptychia hesionides* | Present | Nymphalidae | Satyrinae |
| *Paryphthimoides grimon* | Present | Nymphalidae | Satyrinae |
| *Pedaliodes* sp. | Present | Nymphalidae | Satyrinae |
| *Penthema darlisa* | Absent | Nymphalidae | Satyrinae |
| *Percnodaimon merula* | Present | Nymphalidae | Satyrinae |
| *Pharneuptychia innocentia* | Present | Nymphalidae | Satyrinae |
| *Pierella lamia* | Present | Nymphalidae | Satyrinae |
| *Pindis squamistriga* | Present | Nymphalidae | Satyrinae |
| *Platypthima ornata* | Present | Nymphalidae | Satyrinae |
| *Praepedaliodes* sp. | Absent | Nymphalidae | Satyrinae |
| *Proboscis propylea* | Present | Nymphalidae | Satyrinae |
| *Pronophila thelebe* | Present | Nymphalidae | Satyrinae |
| *Proterebia afra* | Present | Nymphalidae | Satyrinae |
| *Pseudochazara mamurra* | Present | Nymphalidae | Satyrinae |
| *Pseudohaetera hypaesia* | Present | Nymphalidae | Satyrinae |
| *Pseudomaniola phaselis* | Present | Nymphalidae | Satyrinae |
| *Pseudonympha magus* | Present | Nymphalidae | Satyrinae |
| *Punapedaliodes flavopunctata* | Present | Nymphalidae | Satyrinae |
| *Pyronia cecilia* | Present | Nymphalidae | Satyrinae |
| *Quilaphoetosus monachus* | Present | Nymphalidae | Satyrinae |
| *Ragadia makuta* | Present | Nymphalidae | Satyrinae |
| *Rareuptychia clio* | Present | Nymphalidae | Satyrinae |
| *Rhaphicera dumicola* | Present | Nymphalidae | Satyrinae |
| *Satyrodes eurydice* | Present | Nymphalidae | Satyrinae |
| *Satyrus actaea* | Present | Nymphalidae | Satyrinae |
| *Steremnia umbracina* | Absent | Nymphalidae | Satyrinae |
| *Steroma modesta* | Absent | Nymphalidae | Satyrinae |
| *Stichophthalma howqua* | Present | Nymphalidae | Satyrinae |
| *Stygionympha vigilans* | Present | Nymphalidae | Satyrinae |
| *Taenaris cyclops* | Present | Nymphalidae | Satyrinae |
| *Tarsocera fulvina* | Present | Nymphalidae | Satyrinae |
| *Taydebis peculiaris* | Present | Nymphalidae | Satyrinae |
| *Taygetis laches* | Present | Nymphalidae | Satyrinae |
| *Thaumantis klugius* | Present | Nymphalidae | Satyrinae |
| *Thauria aliris* | Present | Nymphalidae | Satyrinae |
| *Tisiphone abeona* | Present | Nymphalidae | Satyrinae |
| *Torynesis magna* | Present | Nymphalidae | Satyrinae |
| *Triphysa phryne* | Present | Nymphalidae | Satyrinae |
| *Xanthotaenia busiris* | Present | Nymphalidae | Satyrinae |
| *Yphthimoides cipoensis* | Present | Nymphalidae | Satyrinae |
| *Ypthima baldus* | Present | Nymphalidae | Satyrinae |
| *Ypthimomorpha itonia* | Present | Nymphalidae | Satyrinae |
| *Zethera incerta* | Present | Nymphalidae | Satyrinae |
| *Zeuxidia dorhni* | Present | Nymphalidae | Satyrinae |
| *Zipaetis saitis* | Present | Nymphalidae | Satyrinae |
| *Zischkaia fumata* | Present | Nymphalidae | Satyrinae |
| *Curetis bulis* | Absent | Lycaenidae | Curetinae |
| *Lycaena helloides* | Absent | Lycaenidae | Lycaeninae |
| *Liphyra brassolis* | Absent | Lycaenidae | Miletinae |
| *Miletus ancon* | Absent | Lycaenidae | Miletinae |
| *Celastrina neglecta* | Absent | Lycaenidae | Polyommatinae |
| *Baliochila minima* | Absent | Lycaenidae | Poritiinae |
| *Poritia erycinoides* | Absent | Lycaenidae | Poritiinae |
| *Lucia limbaria* | Present | Lycaenidae | Theclinae |
| *Thecla coelicolor* | Absent | Lycaenidae | Theclinae |
| *Euselasia orfita* | Present | Riodinidae | Euselasiinae |
| *Styx infernalis* | Absent | Riodinidae | Euselasiinae |
| *Hamearis lucina* | Absent | Riodinidae | Nemeobiinae |
| *Amarynthis meneria* | Absent | Riodinidae | Riodininae |
| *Crocozona coecias* | Absent | Riodinidae | Riodininae |
| *Emesis mandana* | Absent | Riodinidae | Riodininae |
| *Nymphidium onaeum* | Absent | Riodinidae | Riodininae |
| *Riodina lysippus* | Absent | Riodinidae | Riodininae |
| *Aporia crataegi* | Absent | Pieridae | Pierinae |
| *Pieris napi* | Absent | Pieridae | Pierinae |
| *Colias eurytheme* | Present | Pieridae | Coliadinae |
| *Eurema hecabe* | Absent | Pieridae | Coliadinae |
| *Leptidea sinapis* | Absent | Pieridae | Dismorphiinae |
| *Pseudopontia paradoxa* | Absent | Pieridae | Pseudopontiinae |
| *Barona brevicornis* | Absent | Papilionidae | Baroniinae |
| *Graphium agamemnon* | Absent | Papilionidae | Papilioninae |
| *Papilio glaucus* | Absent | Papilionidae | Papilioninae |
| *Papilio machaon* | Absent | Papilionidae | Papilioninae |
| *Troides helena* | Absent | Papilionidae | Papilioninae |
| *Parnassius phoebus* | Present | Papilionidae | Parnassinae |
