# Supplementary material for: A Single Origin for Nymphalid Butterfly Eyespots Followed by Widespread Loss of Associated Gene Expression
Source: PLoS Genet. 2012 Aug 16;8(8):e1002893. doi: 10.1371/journal.pgen.1002893 (PMC3420954; doi:10.1371/journal.pgen.1002893)
Supplement: Table S2 — Model comparisons for the number of eyespot origins in Nymphalidae. Node states refer to ancestral state (0 = eyespots absent and 1 = eyespots present) assigned to nodes as lettered in Figure S1. Differences in log likelihoods are relative to the best-fit model of a single origin, after divergence of Danainae; bold ΔlnL values indicate models providing a significantly worse fit than the single origin model. (DOC) [file pgen.1002893.s008.doc]

**Table S2.** Model comparisons for the number of eyespot origins in Nymphalidae. Node states refer to ancestral state (0 = eyespots absent and 1 = eyespots present) assigned to nodes as lettered in Figure S1. Differences in log likelihoods are relative to the best-fit model of a single origin, after divergence of Danainae; bold ΔlnL values indicate models providing a significantly worse fit than the single origin model.

| Model | Node States | -lnL | ΔlnL |
| --- | --- | --- | --- |
| Single origin, after divergence of Danainae | 0: A  1: B, C, D, E, F, G, H, I, J | 192.6602 | - |
| Single origin, before divergence of Danainae | 0: -  1: A, B, C, D, E, F, G, H, I, J | 194.0897 | 1.4295 |
| Two origins | 0: A, B, I  1: C, D, E, F, G, H, J | 194.9453 | **2.2851** |
| Two origins + loss in Calinaginae | 0: A, B  1: C, D, E, F, G, H, I, J | 195.2213 | **2.5611** |
| Three origins | 0: A, B, C, E, I  1: D, F, G, H, J | 198.1891 | **5.5289** |
| Three origins + loss in Pseudergolinae | 0: A, B, C, I  1: D, E, F, G, H, J | 197.7763 | **5.1161** |
| Three origins + loss in Calinaginae | 0: A, B, C, E  1: D, F, G, H, I, J | 198.4507 | **5.7905** |
| Three origins + losses in Calinaginae & Pseudergolinae | 0: A, B, C  1: D, E, F, G, H, I, J | 198.0386 | **5.3784** |
