# Supplementary material for: A Single Origin for Nymphalid Butterfly Eyespots Followed by Widespread Loss of Associated Gene Expression
Source: PLoS Genet. 2012 Aug 16;8(8):e1002893. doi: 10.1371/journal.pgen.1002893 (PMC3420954; doi:10.1371/journal.pgen.1002893)
Supplement: Table S3 — Model comparisons of expression evolution of en and Antp in eyespot centers. Node states refer to ancestral state (0 = no central expression and 1 = expression in future eyespot centers) assigned to nodes as numbered in Figure S3. Differences in log likelihoods are relative to the best-fit model for each gene (the single origin model in en and the two origin, recent gain in Biblidinae model in Antp); significantly worse models are indicated by bold ΔlnL values. (DOC) [file pgen.1002893.s009.doc]

**Table S3.** Model comparisons of expression evolution of *en* and *Antp* in eyespot centers. Node states refer to ancestral state (0 = no central expression and 1 = expression in future eyespot centers) assigned to nodes as numbered in Figure S3. Differences in log likelihoods are relative to the best-fit model for each gene (the single origin model in *en* and the two origin, recent gain in Biblidinae model in *Antp*); significantly worse models are indicated by bold ΔlnL values.

| Gene | Model | Node States | -lnL | ΔlnL |
| --- | --- | --- | --- | --- |
| *en* | Single origin | 0: 9-12, 20  1: 1-5, 7, 8, 13-19 | 15.9638 | - |
|  | Two origins | 0: 1, 8-12, 20  1: 2-5, 7, 13-19 | 16.5123 | 0.5485 |
|  | Two origins + Loss in Biblidinae | 0: 1, 9-12, 20  1: 2-5, 7, 8, 13-19 | 17.2676 | 1.3038 |
| *Antp* | Single origin | 0: 13-20  1: 1-12 | 11.6623 | **4.9950** |
|  | Two origins: one in Satyrinae and one recent gain in Biblidinae | 0: 1, 8, 9, 11-20  1: 2-7, 10 | 6.6673 | - |
|  | Two origins: one in Satyrinae and one ancient gain in Biblidinae | 0: 1, 8, 11-20  1: 2-7, 9, 10 | 10.6863 | **4.0190** |
|  | Two origins + Loss in Nymphalinae | 0: 1, 13-20  1: 2-12 | 13.8705 | **7.2032** |
